# Direct estimates of absolute ventilation and estimated *Mycobacterium tuberculosis* transmission risk in clinics in South Africa

**DOI:** 10.1371/journal.pgph.0000603

**Published:** 2022-11-02

**Authors:** Peter G. Beckwith, Aaron S. Karat, Indira Govender, Arminder K. Deol, Nicky McCreesh, Karina Kielmann, Kathy Baisley, Alison D. Grant, Tom A. Yates

**Affiliations:** 1 Department of Medicine, University of Cape Town, Cape Town, South Africa; 2 TB Centre, London School of Hygiene & Tropical Medicine, London, United Kingdom; 3 The Institute for Global Health and Development, Queen Margaret University, Edinburgh, United Kingdom; 4 Africa Health Research Institute, School of Laboratory Medicine & Medical Sciences, College of Health Sciences, University of KwaZulu-Natal, Durban, South Africa; 5 Institute of Tropical Medicine, Antwerp, Belgium; 6 Department of Infectious Disease Epidemiology, The London School of Hygiene & Tropical Medicine, London, United Kingdom; 7 School of Laboratory Medicine and Medical Sciences, College of Health Sciences, University of KwaZulu-Natal, Durban, South Africa; 8 Division of Infection and Immunity, Faculty of Medicine, University College London, London, United Kingdom; Zagazig University, Faculty of Medicine, EGYPT

## Abstract

Healthcare facilities are important sites for the transmission of pathogens spread via bioaerosols, such as *Mycobacterium tuberculosis*. Natural ventilation can play an important role in reducing this transmission. We aimed to measure rates of natural ventilation in clinics in KwaZulu-Natal and Western Cape provinces, South Africa, then use these measurements to estimate *Mycobacterium tuberculosis* transmission risk. We measured ventilation in clinic spaces using a tracer-gas release method. In spaces where this was not possible, we estimated ventilation using data on indoor and outdoor carbon dioxide levels. Ventilation was measured i) under usual conditions and ii) with all windows and doors fully open. Under various assumptions about infectiousness and duration of exposure, measured absolute ventilation rates were related to risk of *Mycobacterium tuberculosis* transmission using the Wells-Riley Equation. In 2019, we obtained ventilation measurements in 33 clinical spaces in 10 clinics: 13 consultation rooms, 16 waiting areas and 4 other clinical spaces. Under usual conditions, the absolute ventilation rate was much higher in waiting rooms (median 1769 m^3^/hr, range 338–4815 m^3^/hr) than in consultation rooms (median 197 m^3^/hr, range 0–1451 m^3^/hr). When compared with usual conditions, fully opening existing doors and windows resulted in a median two-fold increase in ventilation. Using standard assumptions about infectiousness, we estimated that a health worker would have a 24.8% annual risk of becoming infected with *Mycobacterium tuberculosis*, and that a patient would have an 0.1% risk of becoming infected per visit. Opening existing doors and windows and rearranging patient pathways to preferentially use better ventilated clinic spaces result in important reductions in *Mycobacterium tuberculosis* transmission risk. However, unless combined with other tuberculosis infection prevention and control interventions, these changes are insufficient to reduce risk to health workers, and other highly exposed individuals, to acceptable levels.

## Introduction

Healthcare facilities bring together infectious and susceptible individuals and are important sites for the transmission of airborne pathogens. This is particularly true for *Mycobacterium tuberculosis (Mtb)* in settings with a high TB burden [1]. Clinic attendees, including vulnerable populations such as people living with HIV, may inhale bioaerosols containing *Mtb* produced by individuals with pulmonary tuberculosis (TB) attending the same facility [1–5]. *Mtb* is an important occupational health concern, with health workers at greater risk than the general population of infection and reinfection and, as a result, developing active TB disease [6,7].

By increasing ventilation rates in indoor congregate spaces, transmission of airborne pathogens, such as *Mtb*, Rubeola virus (measles), and SARS-CoV-2, can be reduced [8–12]. However, ventilation rates are difficult to measure and there are limited data available on ventilation rates in public spaces, such as clinics, in sub-Saharan Africa [13–18].

Natural ventilation plays a key role in low and middle-income settings where the resources and infrastructure needed for mechanical ventilation systems are usually unavailable [19]. Natural ventilation can have comparable or superior performance to mechanical ventilation systems [19]. However, rates will vary with changes in wind speed or direction, and inadequate rates of natural ventilation may occur in poorly designed buildings [20].

We aimed to describe the ventilation of waiting areas, consultation rooms, and other clinical spaces across ten clinics that provide primary healthcare in South Africa. Our ventilation experiments were undertaken as part of an interdisciplinary project called *Umoya omuhle* (meaning “good air” in isiZulu), which used a whole systems approach to understand *Mtb* transmission and TB Infection prevention and control in primary healthcare clinics in South Africa [21,22].

## Methods

### Ethics statement

The *Umoya omuhle* study received ethical approval from the Biomedical Research Ethics Committee of the University of KwaZulu-Natal (ref. BE082/18), the Human Research Ethics Committee of the Faculty of Health Sciences of the University of Cape Town (ref. 165/2018), the Research Ethics Committee of Queen Margaret University (ref. REP 0233), and the Observational/Interventions Research Ethics Committee of the London School of Hygiene & Tropical Medicine (ref. 14872). No data on individuals were captured for this aspect of the project. Consent to undertake these experiments was obtained from facility managers.

### Setting

South Africa has one of the highest incidence of both TB disease and HIV-associated TB in the World [23]. The prevalence of bacteriologically confirmed pulmonary TB in South Africa was 852 (95% CI 679–1026) per 100 000 population in the 2017–19 national prevalence survey [24]. As part of the *Umoya omuhle* project, a TB prevalence study was performed at two clinics in 2018–19. The prevalence of TB in clinic attendees in rural KwaZulu-Natal was 1.0%, which was similar to TB prevalence in the surrounding community [25].

In this study, ventilation experiments were performed in five clinics in KwaZulu-Natal and five in Western Cape province, South Africa, between December 2018 and December 2019. These clinics were built between the 1980s and early 2010s and serve both urban and rural populations. The rural clinics serve an estimated 3000 to 8000 attendees per month and the peri-urban/urban clinics serve 1000 to 30,000 attendees per month (Table 1). They were selected to be broadly representative of clinics in the two provinces with respect to location, size, age of building, and type of clinic. Each clinic had a unique design, though some clinics had several rooms that were identical in their layout. Many clinics had a mixture of permanent and temporary buildings. Temporary buildings included pre-fabricated structures that were used to rapidly expand clinic capacity as the antiretroviral therapy programme was being rolled out in the 2000’s [26].

**Table 1:** Characteristics of facilities (n = 10) and rooms (n = 33) in which ventilation experiments were conducted.

Facility code	Decade built	Location	Estimated monthly headcount, n (1000s)	Type of clinic	Rooms studied (n = 33), n	Rooms where CO2 release experiments performed (n = 26), n	Rooms where rebreathed fraction experiments performed (n = 8), n
KZN1	1990s	Peri-urban	11–14	PHC	5	4	1
KZN2	1980s	Rural	5–8	PHC	7	7	0
KZN3	2000s	Peri-urban	27–30	CHC	3	2	1
KZN4	1980s	Urban	27–30	CHC	2*	2	1
KZN6	2000s	Rural	3–6	PHC	2	2	0
WC1	2010s	Peri-urban	25–28	PHC	3	1	2
WC2	2000s	Urban	1–3	PHC	2	1	1
WC3	1980s	Peri-urban	2–5	PHC	5	4	1
WC5	2010s	Urban	27–30	CHC	2	1	1
WC6	2000s	Peri-urban	25–28	PHC	2	2	0

CHC: Community health clinic; PHC: Primary healthcare clinic.

*Both tracer gas release and rebreathed fraction experiments were performed in the same room.

Measurements were undertaken in spaces where patients either waited or interacted with clinic staff. These included: rooms where patient vital signs were measured; rooms where phlebotomy was performed; formal and informal waiting areas; and consultation rooms. The timing of experiments was agreed in advance with clinic managers.

### Weather

KwaZulu Natal has a subtropical climate, while the Western Cape has a more temperate climate. Data on wind speed and temperature were provided by the South African Weather Services for the period 01 January 2018 to 31 December 2020. These consisted of hourly measurements taken at the nearest weather station to each clinic (median Euclidean distance: 20.5km [range: 5-35km]). Using these data, the wind speed and temperature at the time experiments were undertaken were compared to the distribution observed during typical clinic opening hours (0600–1800) across this three-year period.

### Describing spaces

The dimensions of each room were measured using a Bosch PLR 40R digital laser measure (Bosch, Gerlingen, Germany; accuracy +/-2 mm) and room volumes calculated. The configuration of the windows and doors when the room was in routine use was recorded–these were then used as the ‘usual’ conditions in our experiments.

To summarise these data, window configuration was classified into one of four categories: 1) open (all windows fully open); 2) closed (all windows closed); 3) half or less open; or 4) more than half open. For example, if there were three windows, one closed and two half-open, the windows would be 33% open [(0 + 0.5 + 0.5) / 3], so the windows would be classified as ‘half or less open’. The same classification was used for doors.

### Measuring ventilation

#### Tracer gas release experiments

Our primary means of measuring ventilation was a tracer gas release technique using carbon dioxide (CO_2_; Table A in S1 Text) [19,27]. Ventilation was estimated under both ‘usual’ conditions (the configuration of doors and windows observed when the room was in routine use) and ‘ideal’ conditions (with existing windows and doors maximally open; Table B in S1 Text). Air-conditioners were not switched on during experiments. Standard air-conditioners circulate air and have little effect on room ventilation.

At the start of each experiment, windows and doors were closed and any remaining openings were taped closed with plastic sheeting. CO_2_ monitors were placed at two different central locations within the room (Datalogging Indoor Air Quality Meter– 800050, Sper Scientific, Scottsdale, Arizona, USA; accuracy +/- 75 parts per million [ppm] CO_2_). The concentration of CO_2_ in the room was then increased by releasing CO_2_ fire extinguishers for 5–10 seconds. CO_2_ was mixed with room air using a paddle fan for two minutes, with the aim of achieving a stable and homogeneous CO_2_ concentration of 3,000–10,000 ppm throughout the space. The fan was then turned off and, after five minutes, any plastic sheeting was removed, and the windows and doors were placed in either the usual or ideal configuration. CO_2_ levels were then recorded every second for a period of approximately five minutes. The aim was to perform three experiments under usual conditions, and three under ideal conditions, yielding six CO_2_ decay curves under each set of conditions.

#### Rebreathed fraction approach

Tracer gas release experiments were not possible in spaces that 1) could not be vacated by patients or clinic staff or 2) were large and very open to the outdoors, meaning high levels of CO_2_ could not be attained at baseline. Where tracer gas release experiments were not possible, an established approach [28,29] was adapted to estimate the ventilation rate using data on indoor CO_2_ concentrations, outdoor CO_2_ concentrations, and occupancy [30]. These experiments were undertaken in eight main waiting areas during working hours when spaces were occupied by patients and staff.

In these waiting areas, three CO_2_ monitors were placed at different central locations and one monitor placed immediately outside the building to capture the CO_2_ concentration in the replacement air. Measurements were obtained every second. The number of individuals in the room was counted every ten minutes, categorising individuals as aged <1 year, 1–5 years, or >5 years. Data were collected for between one and three hours under each set of conditions (usual and ideal).

### Statistical analysis

The tracer gas release experiments produced data similar to those presented in Fig A in S1 Text. Analysis focused on the right-hand side of these curves. Specifically, data were included from 30 seconds after the doors and windows were opened until CO_2_ levels reached 200 ppm above baseline or, where no baseline value was available, until CO_2_ levels reached 800 ppm.

Prior to analysis, the CO_2_ data were smoothed, using a sixty second moving average (mean), consisting of the 30 seconds before and after each measurement. Using these smoothed data, the natural logarithms of the CO_2_ concentration measurements were plotted against time in hours. A linear regression line was then fitted through each trace, with the gradient of this line providing an estimate of the number of air changes per hour (ACH). With (typically) six such estimates per experiment (three experiments, two CO_2_ meters per experiment), a pooled estimate of the number of air changes per hour, with an associated 95% confidence interval (CI), was obtained using random effects meta-analysis.

The absolute ventilation rate was then calculated as the product of the number of air changes per hour and the room volume (see Eq 1). In this study, we have presented estimates of the absolute ventilation rate in m^3^/hour. We convert these estimates to litres/second, where needed, to make comparisons with published guidelines.


Q=ACHXVolume (Eq 1)

Q = absolute ventilation (volume/time); ACH = air changes per hour; Volume = room volume

Whilst ACH are commonly mentioned in discussions about ventilation and the transmission of airborne pathogens, the absolute ventilation rate is the ventilation parameter that determines risk of airborne infection. Use of ACH as a measure of ventilation can be misleading, as a small space with a high number of ACH may be less well ventilated than a larger space with fewer ACH. However, we report data on room volumes and ACH, allowing readers to understand what is driving differences in the absolute ventilation rate.

To analyse the paired indoor and outdoor CO_2_ measurements, we developed an adaptation of the Persily and de Jonge method [29] to allow for non-steady state conditions. The outdoor CO_2_ concentration was estimated as a mean obtained through curve fitting, using linear interpolation. The indoor CO_2_ concentration in each space was calculated at each time point by taking the mean value from the three indoor monitors. The number of individuals in each age group in the room at each time point was multiplied by their assumed CO_2_ generation rate to estimate the total CO_2_ generation rate at each time point. Age-specific CO_2_ production rates were taken from Persily and de Jonge [29], assuming an activity rate of 1.2 MET (occupants sitting quietly). A simple linear regression model was then fitted describing how, at each time point, the difference between the indoor CO_2_ concentration and the outdoor CO_2_ concentration related to the total CO_2_ generation rate. The slope of this regression line estimated the absolute ventilation rate. More details of this method are given elsewhere [30]. Note, outdoor CO_2_ concentrations in our experiments were measured and found to be stable. In settings where outdoor CO_2_ levels are more variable, investigators using this method may prefer to update the outdoor CO_2_ measurement at each timestep.

Most spaces were only visited on one occasion and, as such, our estimates of ventilation do not fully capture variation in ventilation rates associated with, for example, changes in wind speed or direction. For this reason, when combining data from different spaces, we opted for a descriptive presentation of our results. We avoided calculating pooled estimates with associated confidence intervals, as we did not wish to overestimate precision.

Estimates of ventilation rates per person were calculated by dividing the absolute ventilation rates by the number of people in the room. For consulting rooms, we used two people (as this was the most frequent observation) and for waiting areas, we used the highest number of people observed during our time at the clinics.

### Estimating transmission risk

We related our calculated absolute ventilation rate to the risk of *Mtb* transmission using the Wells Riley Equation [31] (Eq 2). The Wells-Riley equation models transmission risk for airborne infections as a Poisson process. It takes into account the time spent in a space, the number of infectious individuals present, the number of ‘infectious quanta’ they produce per unit time (usually assumed), the volume of air susceptible individuals breathe per unit time, and the absolute ventilation rate [31]. Here, quanta are defined as ‘the number of infectious airborne particles required to infect which may be one or more airborne particles’ [31]. The Wells-Riley equation assumes a well-mixed airspace so cannot be used to model variation in transmission risk within a room [32].


$$P=1-e^{\left(-\frac{Iqpt}{Q}\right)}$$ (Eq 2)

P = probability for each susceptible individual of being infected over time t; I = Number of infectious individuals; q = number of infectious quanta they produce per hour; p = pulmonary ventilation rate of susceptible individuals (volume/time); t = exposure time; Q = absolute room ventilation (volume/time)

In estimating transmission risk using the Wells-Riley equation, we assumed a single infectious individual was present and that susceptible individuals had a pulmonary ventilation rate of 0.6m^3^/hr. With TB prevalence in these clinics approximately 1% [25] and 1000 to 30,000 attendees per month, 0–10 individuals with viable *Mtb* in sputum might be expected to visit these clinics each day.

The infectiousness of people with pulmonary TB is known to be heterogeneous. The standard assumption is that infectious individuals produce 1.25 infectious quanta per hour, based on data from early studies at Johns Hopkins [33]. A similar study of HIV-positive adults with pulmonary TB in Lima, Peru, reported a mean quanta production rate of 8.2 per hour, and the most infectious individual in that study produced 226 quanta per hour [34]. We, therefore, estimated transmission risk at 1.25, 8.2, and 226 quanta/hr.

We presented estimates of transmission risk in two different ways. First, we used heat maps to describe transmission risk across a range of plausible ventilation rates and visit durations. Second, we used ‘illustrative scenarios’, presenting estimated risk of *Mtb* infection following typical exposures: the risk of a health worker becoming infected during one month at work, and the risk of a patient becoming infected during a typical clinic visit. For the first scenario, we presented the estimated risk of a health worker becoming infected with *Mtb* if, on each of 25 working days, they spent 15 minutes in a consulting room with an individual with infectious pulmonary TB. For the second scenario, we presented the estimated risk of becoming infected with *Mtb* during 2.5 hours, a typical visit duration [35], in a waiting room with one individual with infectious pulmonary TB. In both scenarios, we assumed that neither the infectious individual nor the susceptible individual wore a mask or respirator. Risk was estimated using each of the three quanta production rates and using the ventilation rate for each room type under both usual and ideal conditions. The main estimate used data from the consulting room or waiting room with the median estimated absolute ventilation rate, but we also produced estimates of transmission risk using the best and worst ventilation rates observed under each set of conditions. Note, we did not apply the 226 q/hr quanta production rate to the health worker scenario, as it is implausible that a health worker would encounter 25 such patients in a single month.

### Tools and code

Stata version 14.2 (StataCorp, College Station, Texas, USA) was used to analyse the tracer gas release experiments and to produce the heat maps. This Stata code is available on GitHub (https://github.com/tayates/uo_ventilation). Analysis of paired indoor-outdoor CO_2_ measurements was carried out using R version 3.6.0 [36]. This R code is also available on GitHub (https://github.com/ArminderD/ventilation.git). The data are available via https://datacompass.lshtm.ac.uk/.

## Results

### Setting

Ventilation experiments were performed in 33 clinic spaces between December 2018 and December 2019. Clinic characteristics are detailed in Table 1. None of these spaces were mechanically ventilated. Tracer gas release experiments were performed in 26 different clinic spaces comprising 13 (50%) consulting rooms; nine (35%) waiting areas; and four (15%) other spaces, including corridors, rooms where patients’ vital signs were measured, and phlebotomy rooms. The layout and dimensions of these rooms are described in S2 Text. Eight main waiting areas were assessed using the rebreathed fraction approach. Note, in one waiting area, we performed both tracer gas release and rebreathed fraction experiments.

Under usual conditions in the 26 rooms where the CO_2_ release technique was used, four rooms (15%) had no windows, two (8%) had all existing windows closed, 12 (46%) had their windows less than half open, and eight (31%) had their windows maximally open. All consulting rooms had their doors closed when attending to patients and no waiting rooms had more than half of their doors open when the room was in use (Table 2). There were two consulting rooms with wall mounted air-conditioners.

**Table 2:** The configuration of windows and doors under usual conditions in rooms where tracer gas release experiments were performed (n = 26 experiments on 12 days).

Type of room	Configuration of windows				Configuration of door in rooms with one door (n = 13), n		Configuration of doors in rooms with multiple doors (n = 13), n		
	No windows	All windows closed	Windows open half or less	All windows maximally open	Door closed	Door open	All doors closed	Half or less doors open	Greater than half doors open
Consulting room (n = 13)	0	2	6	5	11	0	0	1	1
Waiting area (n = 9)	3	0	4	2	0	0	0	7	2
Other (n = 4)*	1	0	2	1	1	1	0	0	2
**Total (n = 26)**	**4**	**2**	**12**	**8**	**12**	**1**	**0**	**8**	**5**

*Other rooms included: Two rooms where patient vital signs were measured, one phlebotomy room, one corridor where patients waited.

### Temperature and wind speed

We compared temperature and wind speed measured at the nearest weather station at the time we undertook our experiments to the 3-year mean temperature and wind speed measured during typical clinic opening hours at the same weather station.

**Fig 1:**
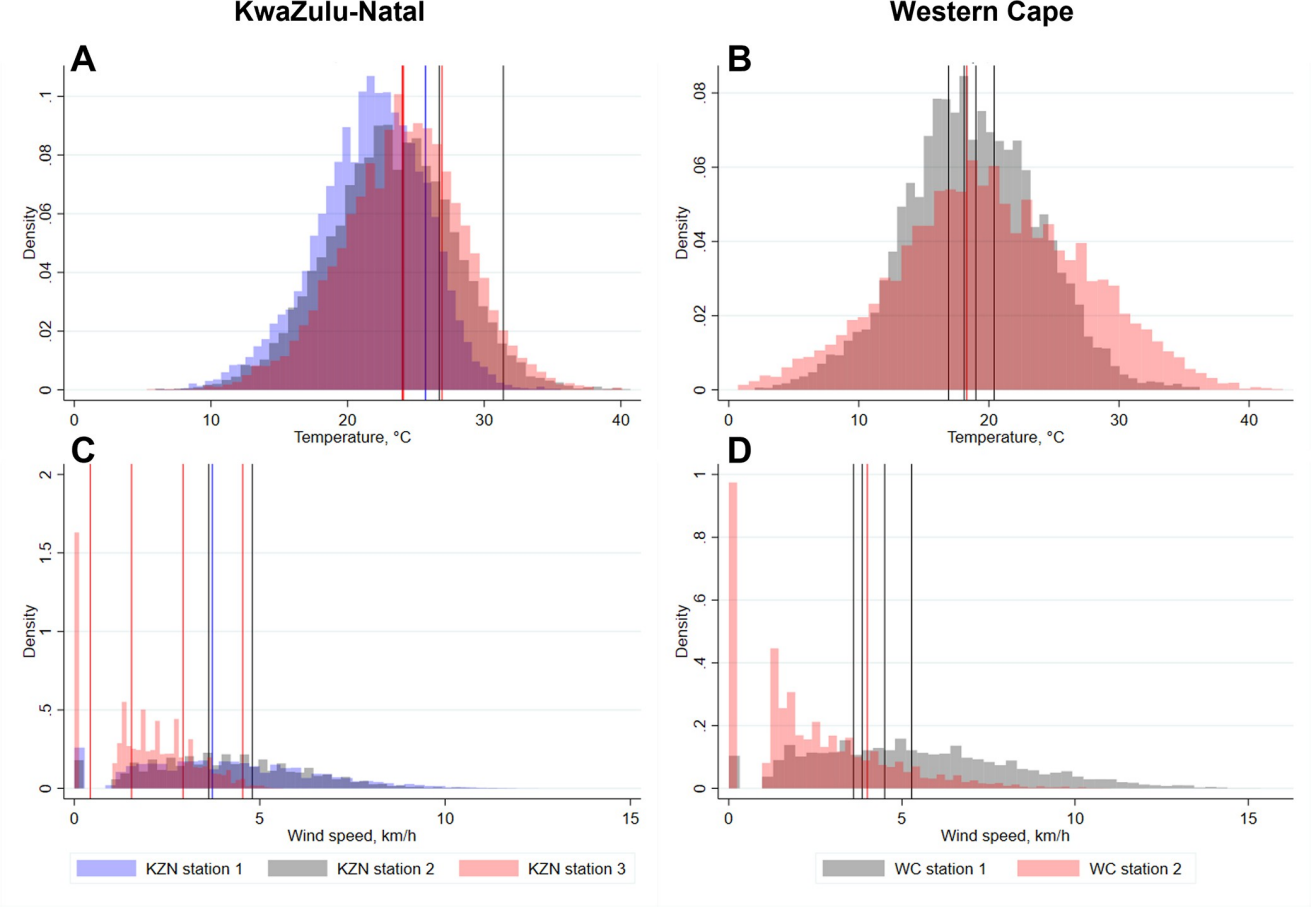
Histograms showing the distribution of temperatures and wind speeds in KwaZulu-Natal and Western Cape during working hours from January 2018–December 2020. Vertical lines show the mean temperatures and wind speeds on the 12 days when tracer gas release experiments were conducted. A: Temperatures in KwaZulu-Natal; B: Temperatures in Western Cape; C: Wind speeds in KwaZulu-Natal; D: Wind speeds in Western Cape. Vertical lines indicate mean temperature or wind speed at the weather station closest to each clinic during working hours on the day the data were collected. Colour of histograms and vertical lines corresponds to specific weather stations in each province. C: Centigrade; km/h: Kilometres per hour; KZN: KwaZulu-Natal; WC: Western Cape.

Temperatures at the times tracer gas release experiments were conducted were within one standard deviations (SD) of the 3-year mean temperature on 10/12 (83%) experiment days and within two SDs on 12/12 (100%) experiment days. Wind speeds were within one and two SDs of the 3-year mean wind speed on 10/12 (83%) and 11/12 (92%) experiment days, respectively (Fig 1).

Temperatures at the time we performed rebreathed fraction experiments were within one and two SDs of the 3-year mean temperature for 7/8 (88%) and 8/8 (100%) experiment days and wind speeds within one and two SDs of the 3-year mean wind speed on 7/8 (88%) and 8/8 (100%) of experiment days (Fig B in S1 Text).

Whilst not far from the seasonal average, the days on which we undertook tracer gas release experiments in KwaZulu-Natal were all towards the warmer end of the distribution.

### Tracer gas release experiments

**Fig 2:**
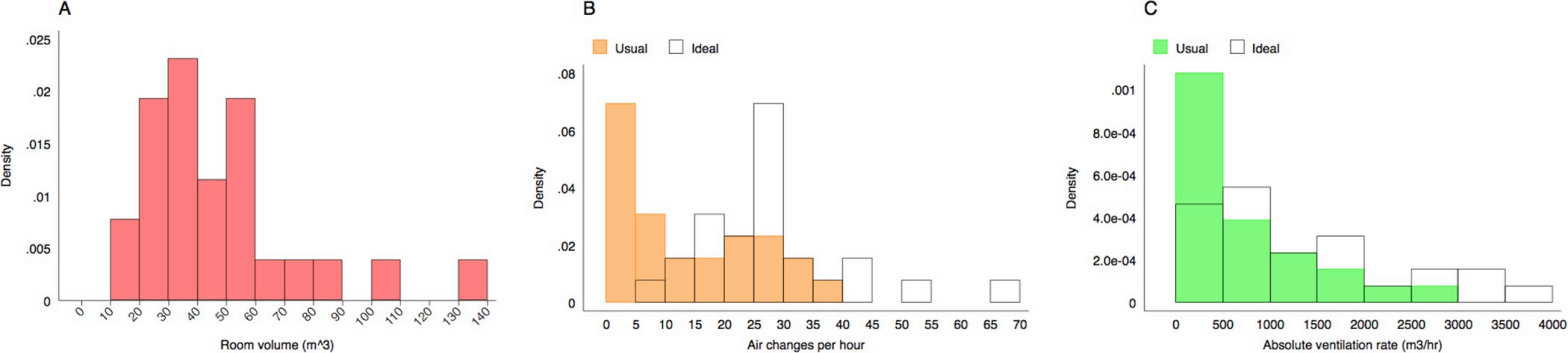
Histograms describing the distribution of room volumes (Fig 2A), the number of air changes per hour (Fig 2B) and the absolute ventilation rate (Fig 2C) in the 26 clinical spaces where we conducted tracer gas release experiments. Ventilation rates are described under usual conditions and ideal conditions (all doors and windows fully open).

**Fig 3:**
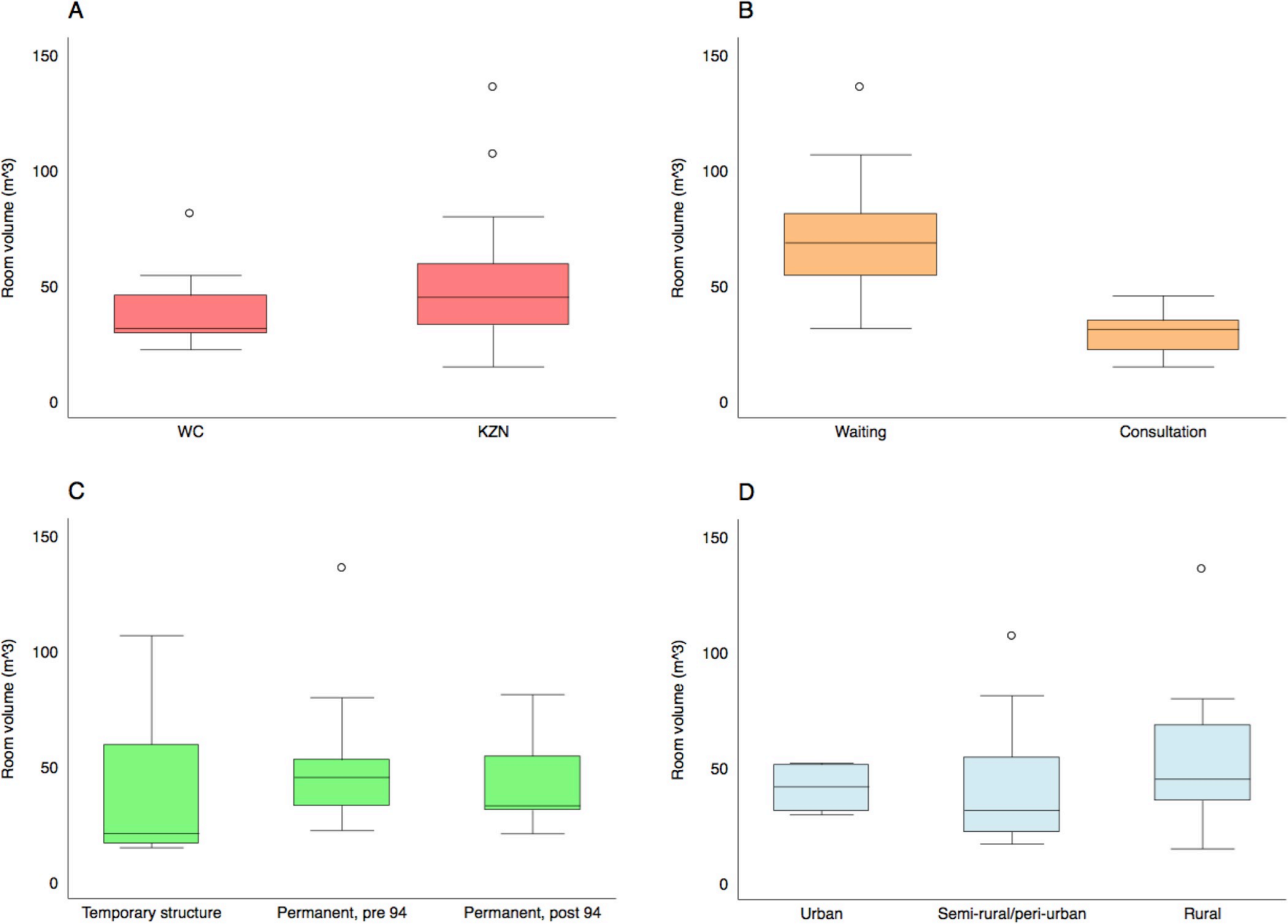
Box and whisker plots describing the distribution of room volumes in the clinical spaces where we conducted tracer gas release experiments. Results are disaggregated by province (Fig 3A), room type (Fig 3B), the age of the building (Fig 3C), and clinic location (Fig 3D). Here, the boxes mark the 25^th^ percentile, the 50^th^ percentile (median) and the 75^th^ percentile, with the whiskers marking the upper and lower adjacent values.

The distribution of room volumes across the 26 spaces in which we performed tracer gas release experiments is presented in Fig 2A. Box and whisker plots in Fig 3 present the same results disaggregated by province, type of room, the age of the building, and whether the community is urban or rural. The distribution of room sizes was skewed with a small number of much larger rooms. As might be expected, consultation rooms (n = 13; median volume 31 m^3^, range 15–45 m^3^) were smaller than waiting rooms (n = 9; median volume 68m^3^, range 31–135 m^3^). Rooms in temporary structures (n = 5; median volume 21 m^3^, range 15–106 m^3^) were smaller than those in permanent structures (n = 21; median volume 43 m^3^, range 21–135 m^3^).

**Fig 4:**
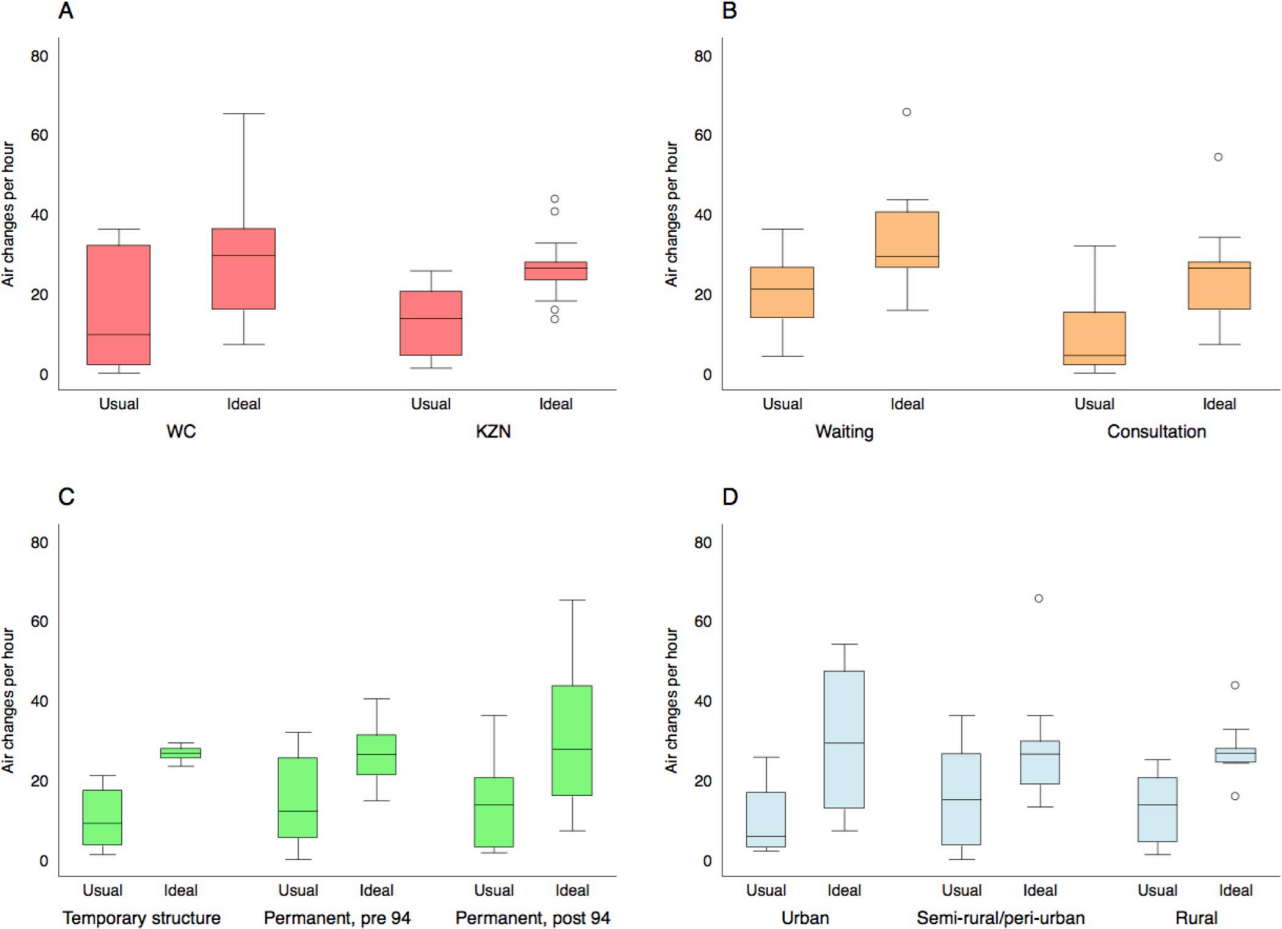
Box and whisker plots describing the distribution of the number of air changes per hour in the 26 clinical spaces where we conducted tracer gas release experiments. Ventilation rates are described under usual conditions and with all doors and windows fully open. Results are disaggregated by province (Fig 4A), room type (Fig 4B), the age of the building (Fig 4C), and clinic location (Fig 4D). Here, the boxes mark the 25^th^ percentile, the 50^th^ percentile (median) and the 75^th^ percentile, with the whiskers marking the upper and lower adjacent values.

The distribution of the number of ACH under both usual and ideal conditions is presented in Fig 2B. The same data disaggregated by province, type of room, the age of the building, and whether the community is urban or rural are presented in Fig 4.

Note, in one consultation room, ‘usual conditions’ were that existing windows and doors were closed leaving no effective ventilation. The tracer gas release experiments performed under these conditions saw CO_2_ levels rise, as air exhaled by investigators accumulated in the room. Fitting a line through these data would have yielded a negative ACH estimate. We therefore assigned this space a ventilation rate of 0 ACH and 0 m^3^/hr.

The number of ACH varied substantially. It was higher in waiting rooms than in consultation rooms (usual conditions: median ACH in waiting rooms = 21, range 4–36; median ACH in consultation rooms = 4, range 0–32) and, as expected, higher with existing doors and windows fully open (ideal conditions: median ACH in waiting rooms = 29, range 16–65; median ACH in consultation rooms = 26, range 7–54). Opening existing doors and windows resulted in a median 2.1-fold increase in the number of air changes per hour (range 1–25.6; note, this range does not include the consultation room with zero effective ventilation under usual conditions).

**Fig 5:**
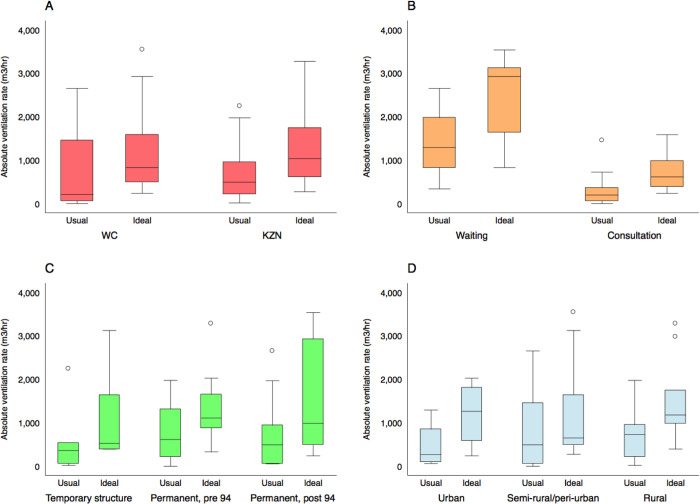
Box and whisker plots describing the distribution of the absolute ventilation rate in the 26 clinical spaces where we conducted tracer gas release experiments. Ventilation rates are described under usual conditions and with all doors and windows fully open. Results are disaggregated by province (Fig 5A), room type (Fig 5B), the age of the building (Fig 5C), and clinic location (Fig 5D). Here, the boxes mark the 25^th^ percentile, the 50^th^ percentile (median) and the 75^th^ percentile, with the whiskers marking the upper and lower adjacent values.

The distribution of the absolute ventilation rates is presented in Fig 2C. These data, disaggregated by province, type of room, the age of the building, and whether the community is urban or rural, are presented in Fig 5. Again, we observed substantial variation. The absolute ventilation rate was much higher in waiting rooms (usual conditions: median absolute ventilation rate = 1289 m^3^/hr, range 338–2651 m^3^/hr) than in consultation rooms (usual conditions: median absolute ventilation rate 197 m^3^/hr, range 0–1451 m^3^/hr). Temporary buildings (usual conditions: median absolute ventilation rate 360 m^3^/hr, range 18–2240 m^3^/hr) were less well ventilated than permanent structures (usual conditions: median absolute ventilation rate 493 m^3^/hr, range 0–2651 m^3^/hr).

The observation that rooms in temporary structures were smaller and had a lower absolute ventilation rate should be treated with caution, as we only obtained measurements in five clinical spaces in temporary structures: 2 waiting rooms, 2 consulting rooms, and one space where vitals were measured.

Table C in S1 Text contains full results for each space in which tracer gas release experiments were performed. Fig C in S1 Text shows the association between wind speed and the absolute ventilation rate for the 26 spaces in which we undertook tracer gas release experiments.

### Rebreathed fraction experiments

**Fig 6:**
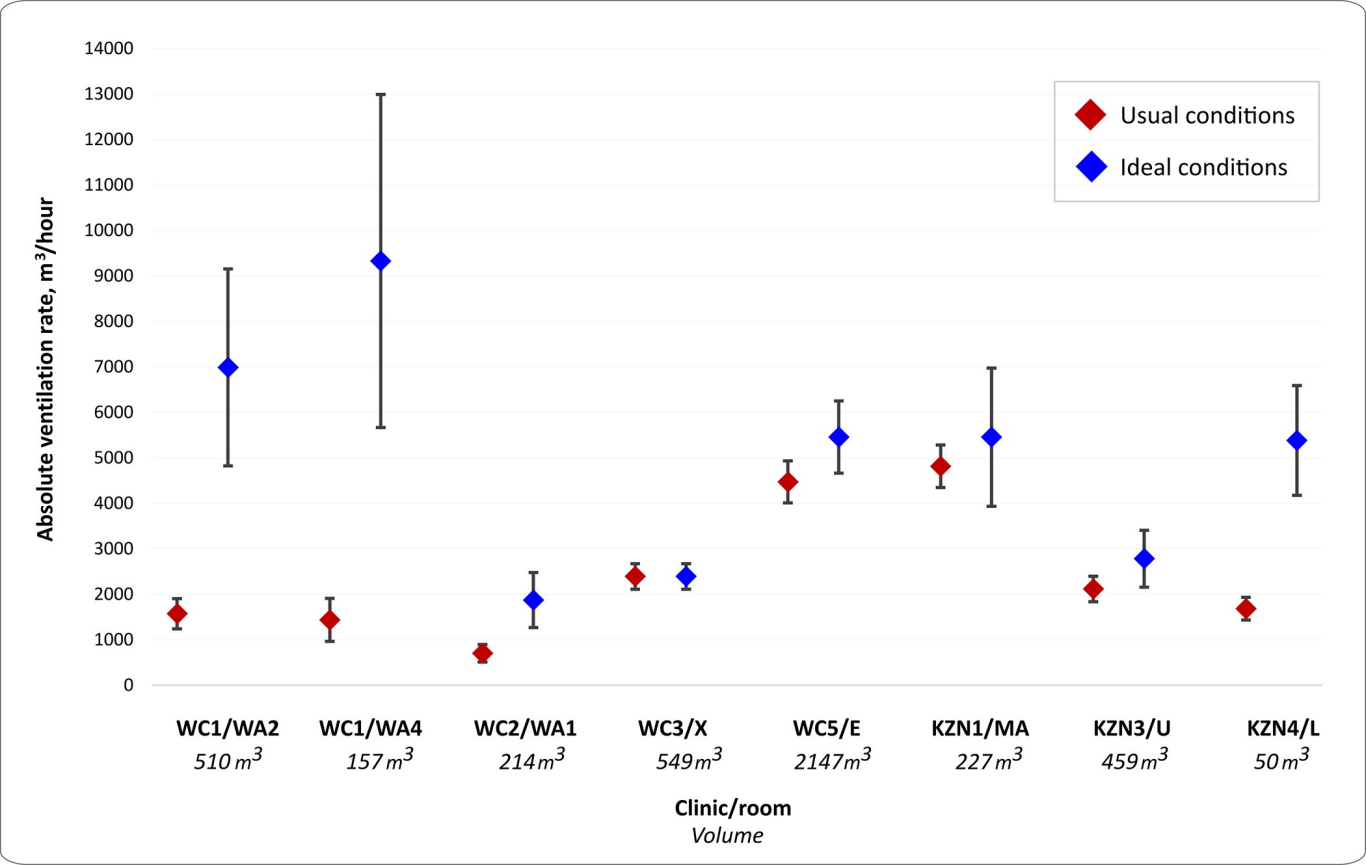
Absolute ventilation rates of eight clinic waiting rooms under usual and ideal conditions, estimated by the rebreathed fraction approach. Usual conditions = configuration of windows and doors observed when the room was in routine use. Ideal conditions = all windows and doors maximally open. Clinic WC3, room X: Usual conditions the same as ideal conditions. Vertical bars indicate upper and lower estimates of 95% confidence intervals WC: Western Cape; KZN: KwaZulu-Natal; WA2, WA4, WA1, X, E, MA, U, L are the codes for the specific waiting rooms where experiments were performed.

The estimated absolute ventilation rates in the eight clinic waiting rooms where the rebreathed fraction approach was used are presented in Fig 6 and Table D in S1 Text. The volume of these waiting rooms was a median 342.7 (range 50.2–2147.3) m^3^. Under usual conditions, there were a median 4.5 ACH (range 2.1–33.5), which improved under ideal conditions to a median 11.2 ACH [range 2.5–107.2]. Absolute ventilation rates were lower under usual conditions than under ideal conditions (median 1898.8 [range 701.5–4815.1] m^3^/hr vs. 5417.8 [range 1870.3–9328.4] m^3^/hr, respectively). Fully opening existing doors and windows improved the absolute ventilation rate by a median 2.0 fold (range 1.0–6.5).

Note, one waiting room (KZN4, Room L) had ventilation estimated using both the tracer gas release and the rebreathed fraction approach. Where results from experiments using both methods are combined, we use the results of the tracer gas release experiments for this space.

### Estimated risk of *Mycobacterium tuberculosis* transmission

**Fig 7:**
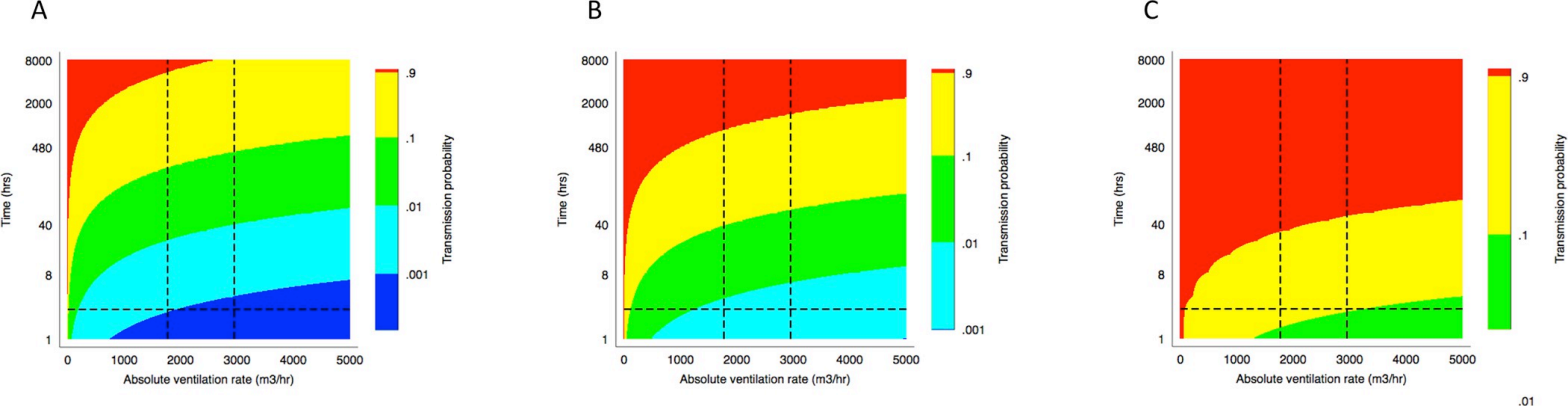
A set of heat maps translating ventilation rate into transmission risk, as estimated using the Wells-Riley equation at 1.25 (A), 8.2 (B) and 226 (C) quanta/hr. The horizontal lines show the median clinic visit duration. The vertical lines show the median absolute ventilation rate in clinic waiting rooms under both usual and ideal conditions.

We related absolute ventilation rate and visit duration to the risk of *Mtb* transmission using the Wells Riley Equation [31], as described above. We produced estimates of infection risk under various assumptions about the quanta production rate (Fig 7). The horizontal lines on the figures give the median clinic visit duration of 2 hours 36 minutes [35]. The vertical lines give the median absolute ventilation rate in waiting rooms under both usual and ideal conditions. Combining data from both the tracer gas release experiments and the rebreathed fraction approach, this figure is 1769 m^3^/hr under usual conditions and 2950 m^3^/hr under ideal conditions.

Assuming a quanta production rate of 1.25 q/hr, that doors and windows were opened to their usual extent, and taking ventilation estimates from the consulting room with the median absolute ventilation rate, the healthcare worker in our illustrative scenario had a 2.3% risk of becoming infected with *Mtb* per month. The equivalent risks in the best and worst ventilated consulting rooms were 0.3% and 100% per month. Under the same assumptions, our illustrative patient had a 0.1% chance of becoming infected per visit, with the equivalent risk in the best and worst ventilated waiting rooms being 0.0% and 0.6% per visit. The risks with doors and windows fully opened were 0.8% per month (range of health worker risk across the different consulting rooms 0.3–2.0%) and 0.1% per visit (range of patient risk across the different waiting rooms 0.0–0.2%). Estimates of transmission risk were very sensitive to assumptions about the quanta production rate. For example, using 226 q/hr, and the median absolute ventilation rate, gave our illustrative patient a 17.6% risk of becoming infected per visit with doors and windows opened to their usual extent. Fully opening existing door and windows reduced this risk to 10.9%. Full results from the illustrative scenarios are presented in Tables E and F in S1 Text.

## Discussion

We observed substantial variation between spaces in rates of natural ventilation. Under usual conditions, using the gold standard tracer gas release method, the median number of ACH was 11.7 and the median absolute ventilation rate was 447 m^3^/hr. There were higher ventilation rates in waiting rooms (median Q = 1289 m^3^/hr) than in consultation rooms (median Q = 197 m^3^/hr). In the eight waiting rooms where we used the rebreathed fraction approach, the median absolute ventilation rate, under usual conditions, was 1898.8 m^3^/hr. We showed that ventilation rates could, on average, be doubled if existing doors and windows were opened.

There are few published estimates of the absolute ventilation rate in naturally ventilated public spaces, including clinical spaces, in high burden settings. In Peruvian hospitals, Escombe and colleagues estimated an absolute ventilation rate of 2477 m^3^/hr in naturally ventilated spaces, and 402 m^3^/hr in mechanically ventilated spaces [19]. In a study of four small clinic rooms in Cape Town, South Africa, Cox and colleagues estimated a median ventilation rate of 487 m^3^/hr in the best ventilated space, but none of these experiments were conducted with both the window and the wind-driven roof turbines open [14].

The latest World Health Organization (WHO) guidelines on TB infection prevention and control (TB IPC), published in 2019, do not contain a recommended minimum ventilation rate for clinical spaces [10]. A 2009 WHO guideline recommended that ‘general wards and outpatient spaces’ in naturally ventilated buildings should aim for a minimum ventilation rate of 60 litres/second/person (216 m^3^/hr/person) [8]. For ‘airborne precaution rooms’ (small rooms for accommodating people who may be infectious) the recommended ventilation rate was 160 litres/second/person on average (576 m^3^/hr/person), with a minimum rate of 80 litres/second/person (288 m^3^/hr/person) when wind speed and direction are not favourable. In high burden settings, the transmission risk in general clinic spaces may be equivalent to that in ‘airborne precaution rooms’, given the high prevalence of undiagnosed TB [25].

Dividing the ventilation rates estimated in this study by the per person rates recommended by the WHO yields estimates of maximum occupancy that are lower than those we observed in these clinics. The median ventilation rate in waiting rooms (combining both measurement approaches) meant only eight people could be accommodated under usual conditions, or 13 under ideal conditions, whilst achieving a ventilation rate of 60 litres/second/person. The median ventilation rate in consultation rooms meant doors and windows needed to be fully opened for a health worker and one patient to be accommodated whilst achieving a ventilation rate of 60 litres/second/person. We undertook detailed analysis of patient flow in nine waiting rooms at three clinics, as part of the *Umoya omuhle* project. The least busy waiting area, in a rural clinic, had a median occupancy of 11 people, whereas the busiest waiting area had a median occupancy of 52 people [35]. Consultation rooms typically accommodate a single health worker plus a patient, though patients are often accompanied by a friend or family member.

We would caution against the use of binary thresholds to determine whether ventilation rates are adequate or not. As is apparent from Fig 7 and from the illustrative scenarios, *Mtb* infection risk depends not only on the ventilation rate but also on the duration of exposure and the infectiousness of the index case. Unacceptable levels of risk are likely with prolonged or repeated exposure, or with shorter exposure to highly infectious individuals. A nurse spending her career working only in ‘adequately’ ventilated spaces may be infected with *Mtb* on multiple occasions unless additional precautions, such as the use of N95 respirators, are taken. This is not to say that improvements in the natural ventilation rate may not prevent infections and consequent disease, just that they may not be sufficient to reduce risk to a level that most individuals would deem acceptable [37]. Furthermore, measurements of room ventilation taken at a single timepoint may give false reassurance, as air flow in naturally ventilated spaces will vary with changes in wind speed and direction.

We showed that natural ventilation can be improved by two-fold by fully opening available windows and doors. However, we observed that only 30% of spaces had their windows fully open and all consulting room doors were closed when consultations were taking place. Major barriers to opening existing doors and windows include concerns regards privacy and thermal comfort [38]. Detailed qualitative research exploring TB IPC related beliefs and behaviours in the same clinics will be presented separately.

Our Wells-Riley estimates of *Mtb* transmission risk suggest that substantial reductions in transmission risk can be achieved with both improvements in natural ventilation and reductions in duration of clinic visits (Fig 7). Using a standard assumption about infectiousness (1.25 q/hr), and using ventilation estimates from the consultation room with the median absolute ventilation rate, the estimated risk of infection in a health worker is 2.3% per month (24.8% annual risk of infection), or 0.8% per month (8.7% annual risk of infection) with existing doors and windows fully opened. This is worryingly high. To put these figures into context, standard methods estimate an annual risk of infection in the general population of 0.5–2% [39] in high-burden countries, with a slightly higher force of infection reported in community surveys in the Western Cape [40–42]. Whilst standard methods may underestimate the extent of community transmission [39], the risk to health workers is clearly much higher. Our calculations are consistent with empirical estimates of the force of infection among South African health workers, which suggest 29% are infected with *Mtb* per year [7]. With shorter durations of exposure and waiting rooms being better ventilated than consulting rooms, risk to patients was lower. However, patients attending at the same time as particularly infectious individuals with pulmonary TB (226 q/hr), using ventilation estimates from the waiting room with the median absolute ventilation rate, had a calculated risk of 17.4% per visit, or 10.9% if existing windows and doors were opened fully.

Estimated transmission risk was much higher using ventilation estimates from less well-ventilated spaces. The substantial variation in ventilation rates observed among spaces within clinics suggest that transmission risk might be reduced by reorganising care so that patients spend more time in better ventilated spaces. Low-cost adaptations to existing structures should also be considered including, where feasible, creating covered outdoor waiting areas. A study of small, poorly-ventilated clinic rooms in Cape Town, South Africa, demonstrated improvements in natural ventilation associated with use of wind driven roof turbines or ‘Whirlybird’ fans [14]. A study in Lima, Peru, measured ventilation before and after making changes to six clinical spaces. These changes ranged in intensity from repairing windows that could not be opened at a cost of USD 25, to building a sheltered outdoor waiting area at a cost of USD 7000. The changes resulted in a median 3.0 fold increase in the ventilation rate [43].

However, as has been argued elsewhere [37], in the absence of other precautions (e.g. consistent use of N95 respirators), very high ventilation rates are needed to protect individuals with prolonged exposure, such as health workers. Where feasible, this might be achieved by moving waiting areas outdoors. The use of upper room germicidal ultraviolet (UVGI) has been advocated where there are constraints on the degree of natural ventilation that can be achieved [38]. This technology is effective at reducing *Mtb* transmission [44,45], and can offer protection on calm days and with doors and windows closed. However, recent experience in South Africa of low-quality installations and poor maintenance led to it falling out of favour [46]. UVGI implementation in resource limited settings is an active area of research and innovative approaches may facilitate easier installation and maintenance [47].

As part of the wider *Umoya omuhle* project, we have modelled the impact of TB IPC interventions implemented in clinics, including improvements in natural ventilation, on *Mtb* transmission both within clinics [48] and in surrounding communities [49]. These interventions are highly cost effective [50]. This work considers the infrastructural, organisational, and behavioural changes needed for such interventions to be sustainable [50]. These results support additional investment in a package of IPC interventions to limit the transmission of *Mtb* and other airborne pathogens. Ideally, these interventions would be introduced in manner that allowed robust estimation of their impact, as has previously been done during the national roll out of major interventions in South African healthcare system [51].

Whilst we only undertook ventilation measurements at ten clinics, these included: both new and old buildings, temporary and permanent structures, in urban and rural clinics across two provinces. We did not include clinics in higher altitude inland parts of South Africa, where the winters can be much colder. CO_2_ release experiments are a robust approach to measuring ventilation, though may overestimate dilutional ventilation if some of the replacement air comes from other occupied spaces (e.g., a waiting area adjoining a consultation room). However, the approach is labour intensive, technically demanding, and not possible in large open spaces or spaces that cannot be emptied of people. For this reason, we obtained ventilation estimates using this approach in a limited number of spaces and, usually, only on a single visit. As such, we will not have fully captured the variability in ventilation that might be expected with changes in weather and season. However, considering the full set of spaces that were studied, we obtained measurements over the course of a calendar year. The days on which we took our measurements were broadly representative with respect to temperature and wind speed, although the days we undertook tracer gas release experiments at clinics in KwaZulu-Natal tended to be warmer than average. Mathematically, the rebreathed fraction approach should give comparable results, though it required assumptions about occupants’ metabolic rate. Direct comparison between the two approaches, in the same space and with the same weather conditions, was not possible, because one method required the space to be occupied and other required it to be empty. It should be noted that while the confidence intervals around the estimates obtained using the rebreathed fraction approach were frequently large, this reflects changes in ventilation rates that occurred over the measurement period–for instance, due to changes in wind speed–as well as the precision of our measurements.

Simpler approaches to estimating the absolute ventilation rate are needed. Several have been proposed, such as simply measuring indoor CO_2_ levels [52], or estimating transmission risk using the ‘rebreathed fraction’ approach described by Rudnick and Milton [28]. However, these approaches do not partition risk into that caused by poor ventilation versus that caused by overcrowding, problems with distinct solutions. A single infectious person may transmit to a single susceptible person in a poorly ventilated space with the low room occupancy only resulting in modest rises in background CO_2_ levels. Furthermore, in our experience, rebreathed fraction measurements may be difficult to interpret where there is rapid flux in levels of occupancy–i.e., in small consultation rooms where patients enter and leave frequently.

Ideally, approaches to estimate absolute ventilation rates are needed that do not depend on costly equipment; that can be performed quickly by clinic staff in occupied spaces with minimal training; and with immediate results to guide risk reduction interventions, such as reducing occupancy or opening windows. A possible approach advocated in WHO documents [8,9] makes a set of simple calculations based on wind speed, which must be measured, and the area of the windows of windows and doors. No validation data are presented, and the method can only be applied in spaces with openings on opposite walls. One alternative might be a simplified tracer gas release experiment, ideally using an inert substance not usually present in room air. This might mean that small volumes of tracer gas could be released with the room in routine use. We wonder whether concentrations might then be measured using a meter adapted to feed data to a smartphone, with a phone application used to interpret the decay curve. Obtaining measurements on still days would yield an approximation of the minimum ventilation rate in these naturally ventilated spaces, which is the parameter of greatest interest when estimating transmission risk.

In conclusion, we observed substantial variation in natural ventilation in clinical spaces in primary healthcare facilities in two provinces of South Africa. The worst ventilated spaces were small rooms where doors and windows had been closed. Opening all existing doors and windows resulted in meaningful improvements in ventilation. However, there remained an unacceptable risk of infection in individuals with longer exposure and in those exposed to particularly infectious index cases. A package of IPC interventions, combining improvements in natural ventilation; reorganising care so patients wait in better ventilated spaces or outdoors; and other approaches, including clinic decongestion; are likely needed to reduce *Mtb* transmission to acceptable levels in these settings. In future work, we plan to model adaptations to clinic structures that maximise ventilation without compromising thermal comfort.

## Supporting information

S1 Text Direct estimates of absolute ventilation rates in primary level clinics in South Africa. Table A: Differences between the two methods used to measure ventilation in the clinics. Table B: Definitions of usual and ideal conditions. Table B: Definitions of usual and ideal conditions. Table C: Absolute ventilation rates of the clinic spaces under usual and ideal conditions using the tracer gas release technique. Table D: Ventilation rates using the rebreathed fraction approach using the continuous measurements under usual and ideal conditions. Table E: Risk of Mycobacterium tuberculosis infection for a healthcare worker spending 15 minutes per day for 25 working days with an infectious individual, calculated using the Wells-Riley equation. The probability of infection was calculated separately for each space using the measured absolute ventilation rates under usual and ideal conditions. We calculated risk using both a standard assumption about infectiousness (1.25 quanta/hr) and using a higher estimate taken from the published literature (8.2 quanta/hr). Table F: Risk of Mycobacterium tuberculosis infection for a patient spending 2.5 hours in a waiting room with an infectious individual, calculated using the Wells-Riley equation. The probability of infection was calculated separately for each space, using the measured absolute ventilation rates under usual and ideal conditions. We used both a standard assumption about infectiousness (1.25 quanta/hr) plus two higher estimates from the published literature (8.2 quanta/hr and 226 quanta/hr). Fig A: an example of a tracer gas release experiment from Room C at KZN2 clinic (experiment 6 Monitor a). Following log transformation, the right hand side of the curve is approximately linear. Fig B: Histograms showing the distribution of temperatures and wind speeds in KwaZulu-Natal and Western Cape during working hours from January 2018 –December 2020. Vertical lines shows the mean temperatures and wind speeds on the 8 days when the rebreathed approach experiments were conducted. Fig C: Association between wind speed and the absolute ventilation rates.
(PDF)

S2 Text Dimensions of room spaces where tracer gas release experiments were performed. (PDF)
