# Supplementary material for: Direct estimates of absolute ventilation and estimated Mycobacterium tuberculosis transmission risk in clinics in South Africa
Source: PLOS Glob Public Health. 2022 Nov 2;2(11):e0000603. doi: 10.1371/journal.pgph.0000603 (PMC10021606; doi:10.1371/journal.pgph.0000603)

S1 Text: Direct estimates of absolute ventilation rates in primary level clinics in South Africa

Supplementary Tables

Table A: Differences between the two methods used to measure ventilation in the clinics

**Name of method**	**Brief description of method**	**Room conditions in which experiments performed**	**Occupancy of space at time of experiment**	**Rooms in which experiments were performed in**	**Advantages**	**Disadvantages**
Tracer gas release experiments	CO_2 was used as the tracer gas for this method. CO_2 was released. The windows and doors were opened and the decay of the CO_2 was measured.	Ideal and usual conditions	Rooms had no HCWs or patients in the room	Mainly small spaces where a high CO_2 baseline could be achieved	Gold standard	CO_2 is released into the atmosphere<br><br>Difficult to measure large spaces with this method<br><br>Difficult to perform during work hours in areas with a large occupancy
Rebreathed fraction approach	CO_2 levels were measured every second in three inside sites of the waiting room and at one site outside of the building. Headcounts were performed.	Ideal and usual conditions	Rooms were occupied by HCWs and patients	Main waiting rooms	Can be performed during working hours.	Headcounts were performed every 10 minutes. The waiting rooms were very dynamic so the variations of CO_2 produced by the number of people may not have been captured

CO_2: Carbon dioxide HCW: healthcare workers

Table B: Definitions of Usual and Ideal conditions

Term	Description
Usual conditions	The configuration of the existing windows and doors in the room observed on the day of room volume measurements (in the case of the tracer gas release experiments); or<br>The configuration of the existing windows and doors found when in use (in the case of the rebreathed fraction approach)
Ideal conditions	All windows and doors fully opened to allow for maximal ventilation

Table C: Absolute ventilation rates of the clinic spaces under usual and ideal conditions using the tracer gas release technique

Clinic	Room	Use of room	Room volume (m^3)	ACH under usual conditions (95% CI)	Absolute ventilation rate in m^3/hr under usual conditions (95% CI)	ACH under ideal conditions (95% CI)	Absolute ventilation rate in m^3/hr under ideal conditions (95% CI)	Fold increase in ventilation rate (ideal/usual)
WC1	Room CR3	Consulting room	30.9	1.7 (1.1 – 2.3)	52.4 (34.6 – 70.3)	15.8 (12.4 – 19.2)	489.4 (384.7 – 594.2)	9.3
WC2	Room CR	Consulting room	29.4	2.1 (1.4 –2.8)	62.0 (40.7 – 83.4)	54.0 (44.8 – 63.2)	1586.5 (1316.3 – 1856.7)	25.6
WC3	Room D	Consulting	22.4	0	0	14.7 (12.4 – 17.1)	329.7 (277.5 – 381.9)	*
WC3	Room G	Waiting room	31.3	26.5 (18.0 – 34.9)	827.6 (563.6 – 1091.5)	26.5 (18.0 – 34.9)	827.6 (563.6 – 1091.5)	1
WC3	Room H	Consulting room	45.5	31.9 (25.9 – 38.0)	1451.5 (1176.0 – 1726.9)	34.1 (28.4 – 39.8)	1550.3 (1289.3 – 1811.3)	1.1
WC3	Room J	Consulting room	22.1	9.7 (8.5 – 10.8)	213.4 (188.4 – 238.3)	29.5 (17.3 – 41.7)	651.5 (381.5 – 921.5)	3.1
WC5	Room A	Consulting room	32.9	3.91 (2.4 – 5.5)	128.5 (79.2 – 177.9)	7.2 (5.3 – 9.1)	237.4 (175.1 – 299.6)	1.9
WC6	Room PD	Waiting area	54.3	36.18 (27.2 – 45.2)	1962.9 (1476.4 – 2449.5)	65.2 (53.5 – 76.8)	3535.1 (2901.9 – 4168.3)	1.8
WC6	Room TB	Waiting area	81.0	32.74 (29.2 – 36.3)	2651.0 (2364.5 – 2937.4)	36.1 (25.7 – 46.5)	2923.2 (2081.7 – 3764.6)	1.1
KZN1	Room CD	Vitals	16.7	3.47 (3.1 – 3.9)	58.03 (51.3 – 64.7)	23.4 (18.2 – 28.6)	391.3 (303.7 – 478.8)	6.7
KZN1	Room CI	Consulting	20.8	17.3 (15.0 – 19.7)	360.4 (311.7 – 409.2)	25.4 (22.3 – 28.6)	528.6 (463.0 – 594.2)	1.5
KZN1	Room ME	Consulting	20.7	3.1 (1.2 – 5.01)	63.3 (22.9 – 103.8)	13.2 (12.6 – 13.8)	273.0 (261.4 – 284.7)	4.3
KZN1	Room MF	Consulting	32.8	15.1 (14.2 – 15.9)	493.81 (466.3 – 521.4)	18.8 (17.4 – 20.2)	614.8 (568.4 – 661.1)	1.3

KZN2	Room A	Waiting area	135.4	14.6 (12.0 – 17.1)	1972.7 (1628.0 – 2317.3)	24.2 (18.6 – 29.8)	3273.2 (2511.3 – 4035.2)	1.7
KZN2	Room C	Vitals	42.7	22.3 (20.8 – 23.7)	949.92 (888.0 – 1011.8)	24.3 (23.3 – 25.3)	1036.7 (995.4 – 1077.9)	1.1
KZN2	Room HI	Consulting	34.6	6.4 (3.6 – 9.3)	222.7 (124.6 – 320.7)	26.9 (23.0 – 30.8)	930.3 (794.2 – 1066.4)	4.2
KZN2	Room K	Waiting area	79.6	4.25 (3.2 – 5.3)	338.0 (253.8 – 422.3)	15.7 (11.5 – 20.0)	1252.8 (917.1 – 1588.4)	3.7
KZN2	Room M	Consulting	44.8	4.4 (4.0 – 4.8)	197.4 (179.0 – 215.8)	26.3 (23.6 – 29.1)	1179.3 (1056.7 – 1302.0)	6.0
KZN2	Room S	Consulting	14.7	1.2 (0.8 – 1.7)	18.3 (11.5 – 25.1)	26.7 (23.2 – 30.2)	392.0 (339.9- 444.0)	21.5
KZN2	Room V	Corridor inside	53.4	25.0 (19.7 – 30.4)	1337.1 (1051.2 – 1622.9)	32.6 (19.4 – 45.9)	1743.7 (1035.9 – 2451.6)	1.3
KZN3	Room HAST	Waiting area	106.4	21.1 (17.8 – 24.3)	2240.3 (1894.2 – 2586.4)	29.3 (26.7 – 32.0)	3119.7 (2839.6 – 3399.8)	1.4
KZN3	Room TB	Waiting area	28.1	9.1 (7.9 – 10.3)	255.4 (220.9 – 289.9)	27.7 (23.2 – 32.2)	7778.5 (652.0 – 905.0)	3.1
KZN4	Area L	Main indoor waiting area	50.2	25.7 (23.0 – 28.4)	1289.4 (1156.0 – 1422.7)	40.3 (34.3 – 46.4)	2023.7 (1719.1 – 2328.3)	1.6
KZN4	Space STU	Bloods room	51.8	7.7 (6.6 – 8.9)	400.3 (342.2 – 458.5)	18.1 (15.3 – 20.9)	937.0 (792.0 – 1081.9)	2.3
KZN6	Room KJ	Waiting area	68.3	13.7 (12.1 – 15.3)	936.9 (826.9 – 1046.9)	43.6 (39.4 – 47.7)	2976.7 (2692.9 – 3260.4)	3.2
KZN6	Room N	Consulting room	35.5	20.4 (20.0 – 20.7)	723.7 (710.5 – 736.9)	27.7 (26.3 – 29.2)	985.5 (933.7 – 1037.3)	1.4

*Unable to calculate fold increase as ACH under usual conditions was 0.

Table D: Ventilation rates using the rebreathed fraction approach using continuous measurements under usual and ideal conditions

Clinic	Room	Volume of room (m^3)	ACH under usual conditions (95% CI)	Absolute ventilation rate in m^3/hr under usual conditions (95% CI)	ACH under ideal conditions (95% CI)	Absolute ventilation rate in m^3/hr under ideal conditions (95% CI)	Fold increase in ventilation (ideal/usual)
WC1	Room WA2	509.6	3.1 (2.4 – 3.7)	1572.2 (1238.7 – 1905.6)	13.7 (9.5 –18.0)	6986.8 (4821.6 – 9152.0)	4.4
	Room WA4	157.3	9.1 (6.1 – 12.1)	1436.9 (963.4 – 1910.5)	59.3 (36.0 – 82.6)	9328.4 (5665.7 – 12991.0)	6.5
WC2	Room WA1	213.9	3.3 (2.4 – 4.2)	701.5 (507.5 – 895.4)	8.7 (5.9 – 11.6)	1870.3 (1264.5 – 2476.2)	2.7
WC3	Room X	549.0	4.4 (3.9 – 4.9)	2391.5 (2210.9 – 2672.0)	4.4 (3.8 – 4.9)	2391.5 (2210.9 – 2672.0)	1
WC5	Room E	2147.3	2.1 (1.9 – 2.3)	4470.3 (4007.5 – 4933.0)	2.5 (2.2 – 2.9)	5458.8 (4664.1 – 6253.5)	1.2
KZN1	Room MA	226.7	21.2 (19.2 – 23.3)	4815.1 (4348.4 – 5281.9)	24.1 (17.4 – 30.8)	5454.6 (3933.1 – 6976.2)	1.1
KZN3	Room U	458.7	4.6 (4.0 – 5.2)	2116.9 (1834.7 – 2399.2)	6.1 (4.7 – 7.4)	2782.0 (2155.8 – 3408.3)	1.3
KZN4	Space L	50.2	33.5 (28.5 – 38.5)	1680.59 (1431.1 – 1930.1)	107.2 (83.2 – 131.3)	5381.1 (4172.9 – 6589.2)	3.2

Table E: Risk of Mycobacterium tuberculosis infection for a healthcare worker spending 15 minutes per day for 25 working days with an infectious individual, calculated using the Wells-Riley equation. The probability of infection was calculated separately for each space using the measured absolute ventilation rates under usual and ideal conditions. We calculated risk using both a standard assumption about infectiousness (1.25 quanta/hr) and using a higher estimate taken from the published literature (8.2 quanta/hr).

Facility	Method used to calculate absolute ventilation rates	Consulting room	Risk of becoming infected (1.25 q/hr, usual conditions) (%)	Risk of becoming infected (1.25 q/hr, ideal conditions) (%)	Risk of becoming infected (8.2 q/hr, usual conditions) (%)	Risk of becoming infected (8.2 q/hr, ideal conditions) (%)
WC1	Tracer gas release	Room CR3	8.6	1.0	44.4	6.1
WC2	Tracer gas release	Room CR	7.2	0.3	39.1	1.9
WC3	Tracer gas release	Room D	100	1.4	100	8.9
WC3	Tracer gas release	Room H	0.3	0.3	2.1	2.0
WC3	Tracer gas release	Room J	2.2	0.7	13.4	4.6
WC5	Tracer gas release	Room A	3.6	2.0	21.3	12.2
KZN1	Tracer gas release	Room CI	1.3	0.9	8.2	5.7
KZN1	Tracer gas release	Room ME	7.1	1.7	38.5	10.7
KZN1	Tracer gas release	Room MF	0.9	0.8	6.0	4.9
KZN2	Tracer gas release	Room HI	2.1	0.5	12.9	3.3
KZN2	Tracer gas release	Room M	2.3	0.4	14.4	2.6
KZN2	Tracer gas release	Room S	22.6	1.2	81.4	7.5
KZN6	Tracer gas release	Room N	0.6	0.5	4.2	3.1

Table F: Risk of Mycobacterium tuberculosis infection for a patient spending 2.5 hours in a waiting room with an infectious individual, calculated using the Wells-Riley equation. The probability of infection was calculated separately for each space, using the measured absolute ventilation rates under usual and ideal conditions. We used both a standard assumption about infectiousness (1.25 quanta/hr) plus two higher estimates from the published literature (8.2 quanta/hr and 226 quanta/hr).

**Clinic**	**Method used to calculate absolute ventilation rates**	**Waiting room**	**Risk of becoming infected (1.25 q/hr, usual conditions) (%)**	**Risk of becoming infected (1.25 q/hr, ideal conditions) (%)**	**Risk of becoming infected (8.2 q/hr, usual conditions) (%)**	**Risk of becoming infected (8.2 q/hr, ideal conditions) (%)**	**Risk of becoming infected (226 q/hr, usual conditions) (%)**	**Risk of becoming infected (226 q/hr, ideal conditions) (%)**
WC1	Rebreathed fraction	Room WA2	0.1	0.03	0.8	0.2	19.4	4.7
	Rebreathed fraction	Room WA4	0.1	0.02	0.9	0.1	21.0	3.6
WC2	Rebreathed fraction	Room WA1	0.3	0.1	1.7	0.7	38.3	16.6
WC3	Rebreathed fraction	Room X	0.08	0.1	0.5	0.5	13.2	13.2
WC5	Rebreathed fraction	Room E	0.04	0.03	0.3	0.2	7.3	6.0
KZN1	Rebreathed fraction	Room MA	0.04	0.03	0.3	0.2	6.8	6.0
KZN3	Rebreathed fraction	Room U	0.1	0.1	0.6	0.4	14.0	11.5
WC3	Tracer gas release	Room G	0.2	0.2	1.5	1.5	33.6	33.6
WC6	Tracer gas release	Room PD	0.1	0.1	0.6	0.3	15.9	9.1
WC6	Tracer gas release	Room TB	0.1	0.1	0.5	0.7	12.0	10.9
KZN2	Tracer gas release	Room A	0.1	0.1	0.6	0.4	15.8	9.8
KZN2	Tracer gas release	Room K	0.6	0.1	3.6	1.0	63.3	23.7
KZN3	Tracer gas release	Room HAST	0.3	0.1	0.5	0.4	14.0	10.3
KNZ3	Tracer gas release	Room TB	0.1	0.1	2.3	0.7	46.7	18.7
KZN4	Tracer gas release	Area L	0.1	0.1	0.9	0.6	23.1	15.4
KZN6	Tracer gas release	Room KJ	0.2	0.1	1.3	0.4	30.4	10.8

Figures

Fig A: An example of a tracer gas release experiment from Room C at KZN2 clinic (experiment 6 Monitor A). Following log transformation, the right hand side of the curve is approximately linear.

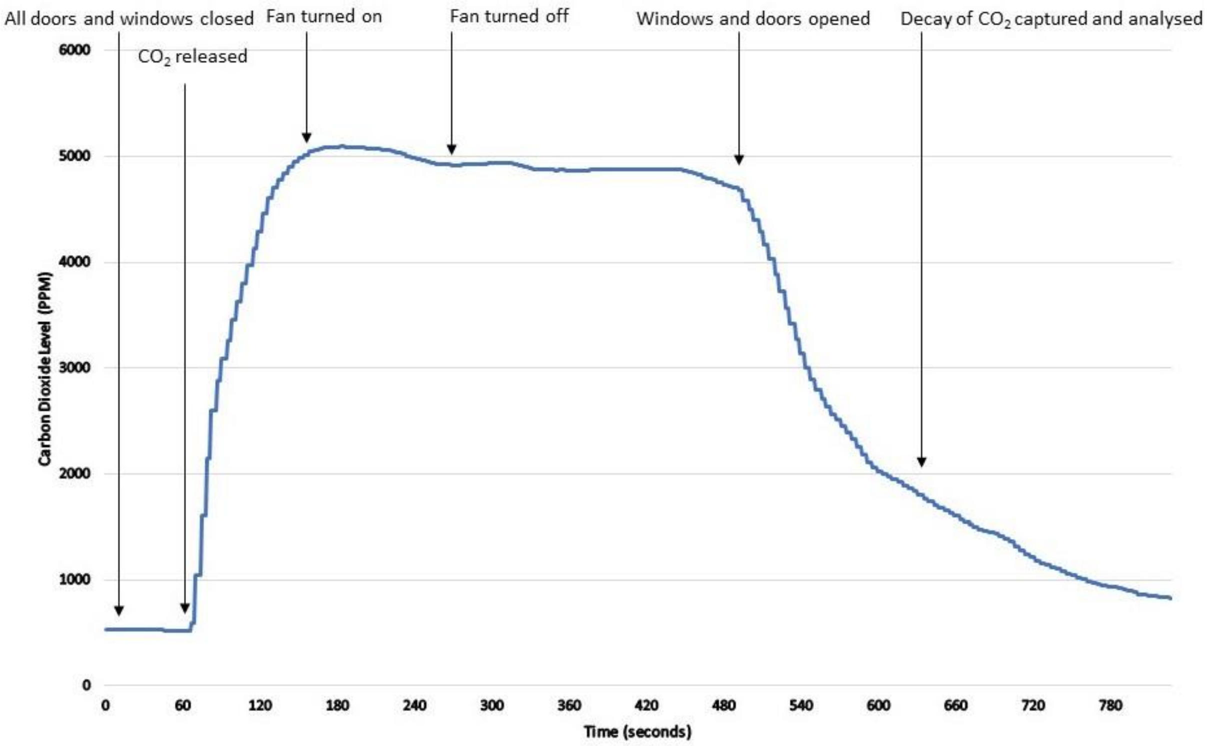

CO_2: carbon dioxide; PPM: parts per million

Fig B: Histograms showing the distribution of temperatures and wind speeds in KwaZulu-Natal and Western Cape during working hours from January 2018 - December 2020. Vertical lines shows the mean temperatures and wind speeds on the 8 days when the rebreathed approach experiments were conducted

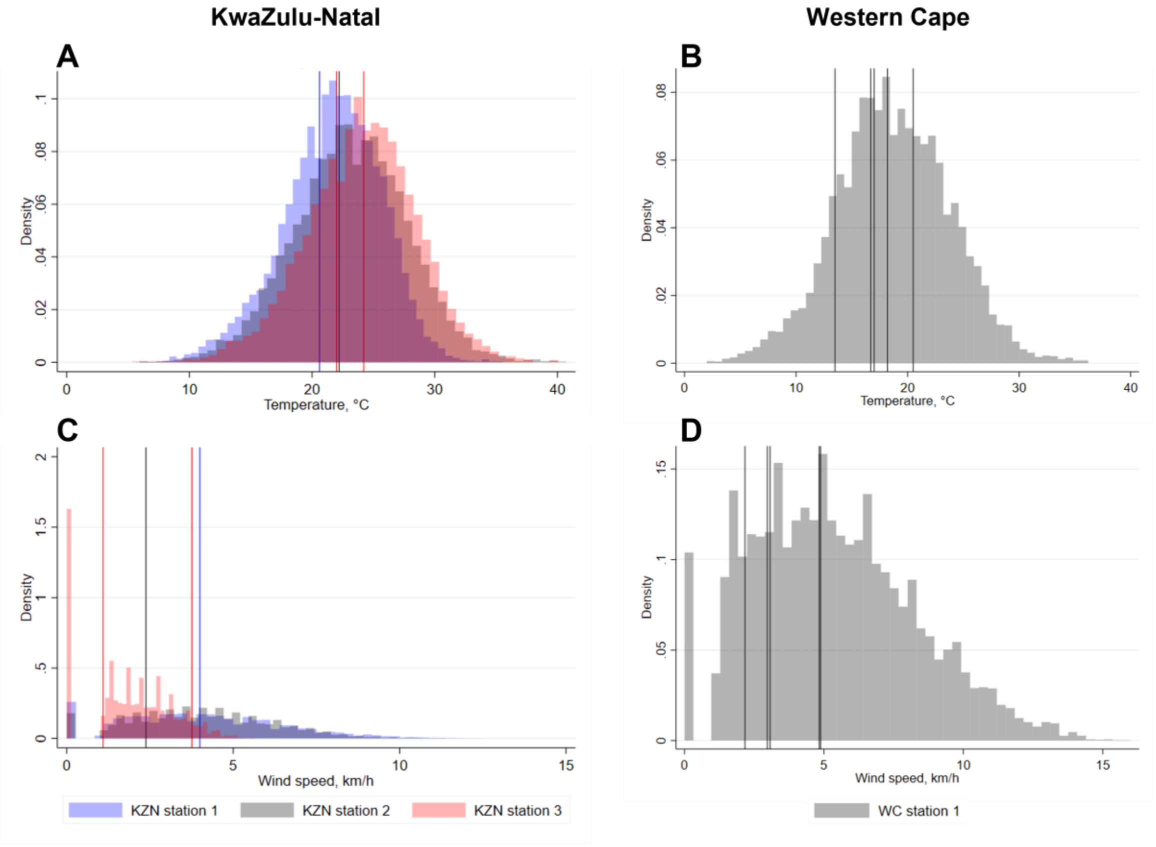

Fig C: The association between wind speed at the nearest weather station and the number of air changes per hour in 26 clinical spaces. Measurements were taken both under usual conditions (Beta: 1.56, 95% CI [-1.98 – 5.10]; R2: 0.03) and ideal conditions (Beta: 3.96, 95 CI [0.23 – 7.70]; R2: 0.17)

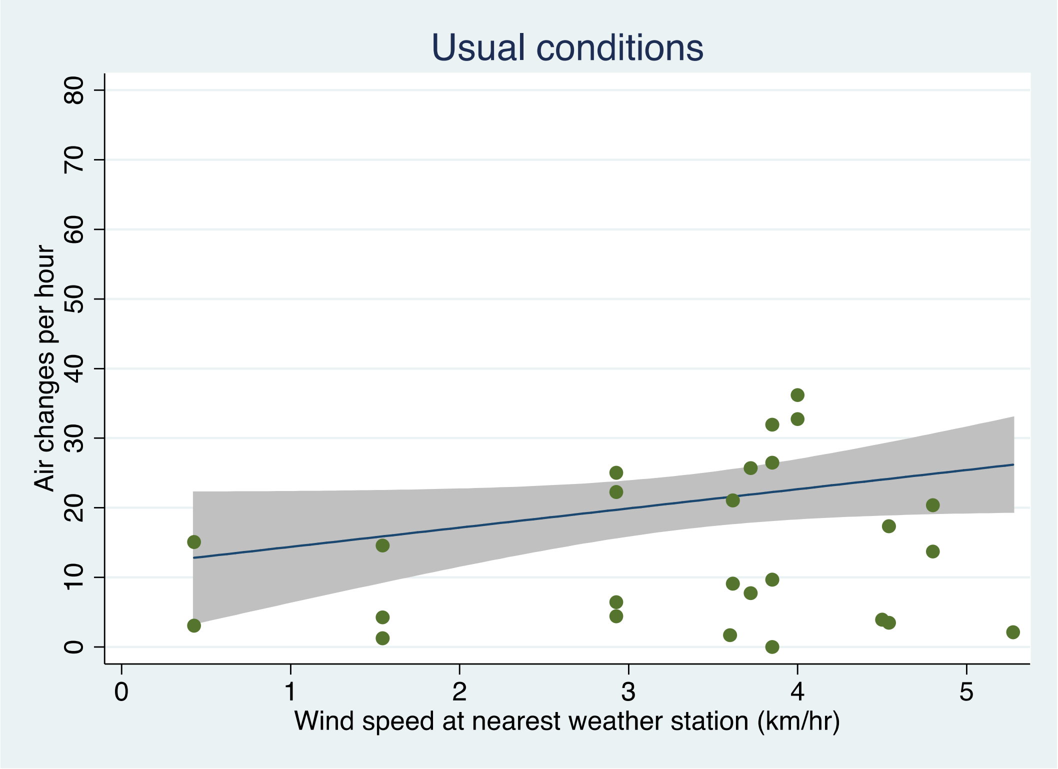

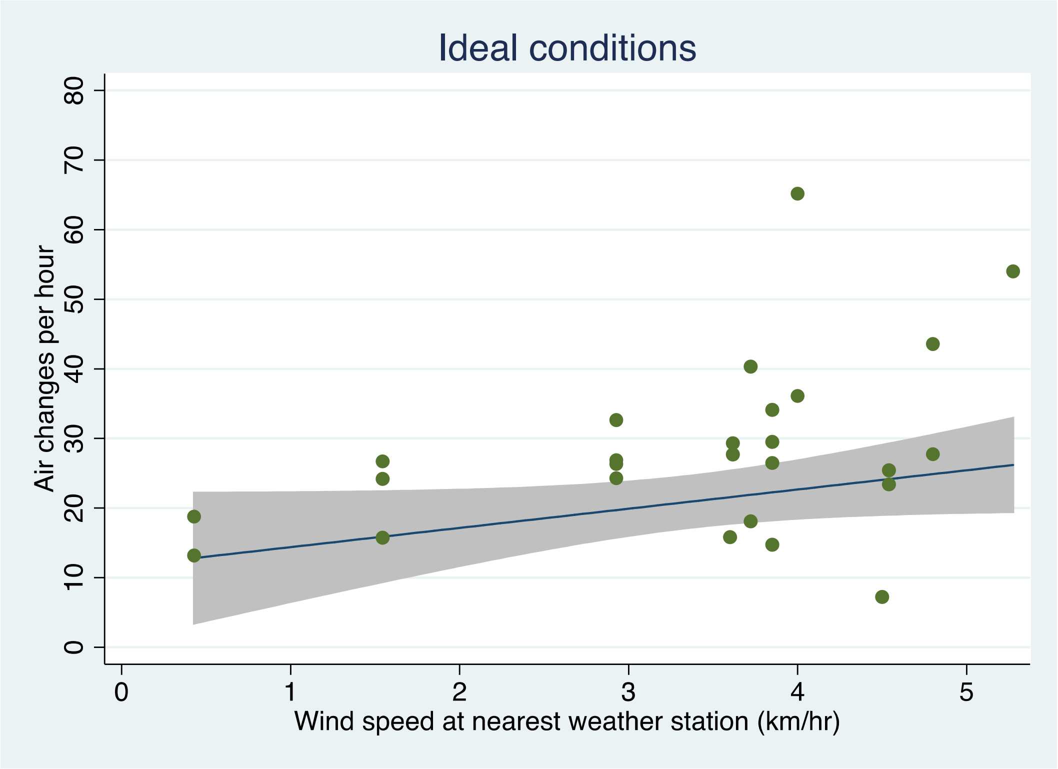

Supplement: S1 Text — Table A: Differences between the two methods used to measure ventilation in the clinics. Table B: Definitions of usual and ideal conditions. Table B: Definitions of usual and ideal conditions. Table C: Absolute ventilation rates of the clinic spaces under usual and ideal conditions using the tracer gas release technique. Table D: Ventilation rates using the rebreathed fraction approach using the continuous measurements under usual and ideal conditions. Table E: Risk of Mycobacterium tuberculosis infection for a healthcare worker spending 15 minutes per day for 25 working days with an infectious individual, calculated using the Wells-Riley equation. The probability of infection was calculated separately for each space using the measured absolute ventilation rates under usual and ideal conditions. We calculated risk using both a standard assumption about infectiousness (1.25 quanta/hr) and using a higher estimate taken from the published literature (8.2 quanta/hr). Table F: Risk of Mycobacterium tuberculosis infection for a patient spending 2.5 hours in a waiting room with an infectious individual, calculated using the Wells-Riley equation. The probability of infection was calculated separately for each space, using the measured absolute ventilation rates under usual and ideal conditions. We used both a standard assumption about infectiousness (1.25 quanta/hr) plus two higher estimates from the published literature (8.2 quanta/hr and 226 quanta/hr). Fig A: an example of a tracer gas release experiment from Room C at KZN2 clinic (experiment 6 Monitor a). Following log transformation, the right hand side of the curve is approximately linear. Fig B: Histograms showing the distribution of temperatures and wind speeds in KwaZulu-Natal and Western Cape during working hours from January 2018 –December 2020. Vertical lines shows the mean temperatures and wind speeds on the 8 days when the rebreathed approach experiments were conducted. Fig C: Association between wind speed an [file pgph.0000603.s001.pdf]
