# Supplementary material for: Direct estimates of absolute ventilation and estimated Mycobacterium tuberculosis transmission risk in clinics in South Africa
Source: PLOS Glob Public Health. 2022 Nov 2;2(11):e0000603. doi: 10.1371/journal.pgph.0000603 (PMC10021606; doi:10.1371/journal.pgph.0000603)
Supplement: S2 Text — (PDF) [file pgph.0000603.s002.pdf]

S2 Text: Dimensions of room spaces where tracer gas release experiments were performed

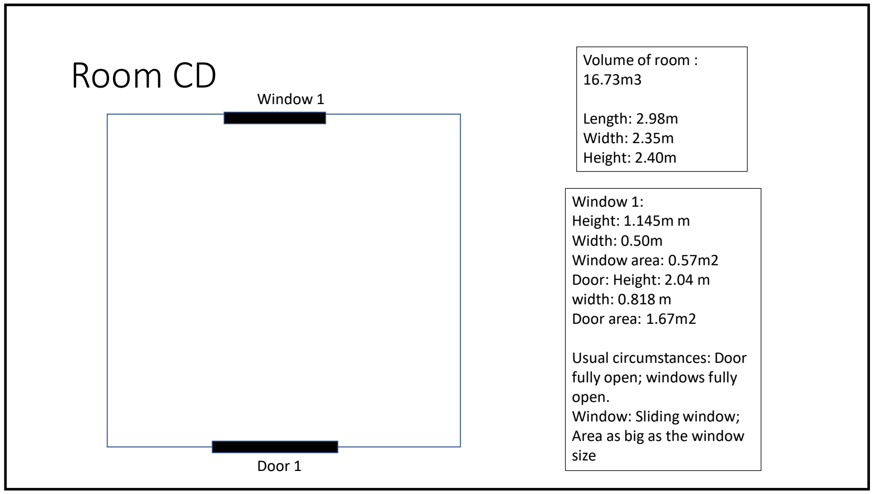

1

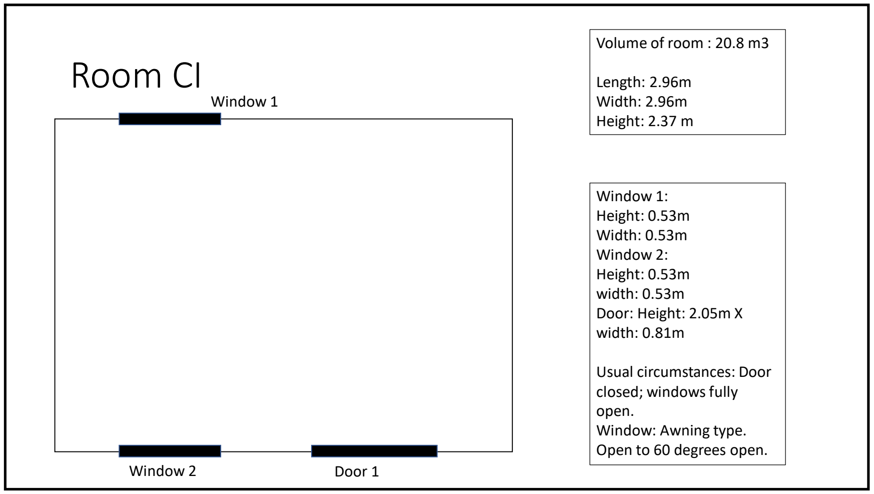

2

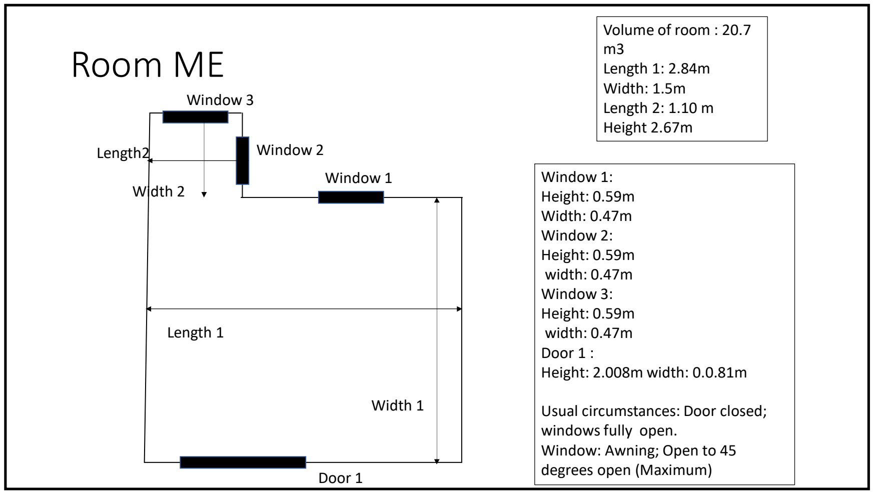

3

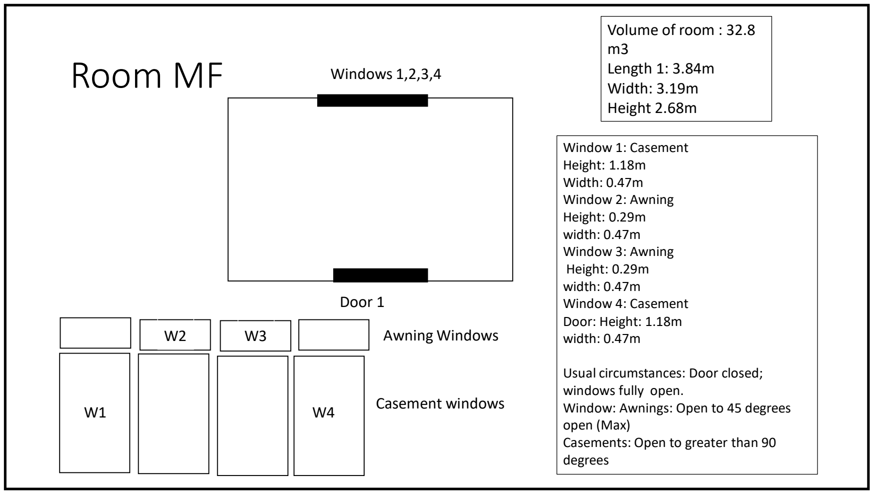

4

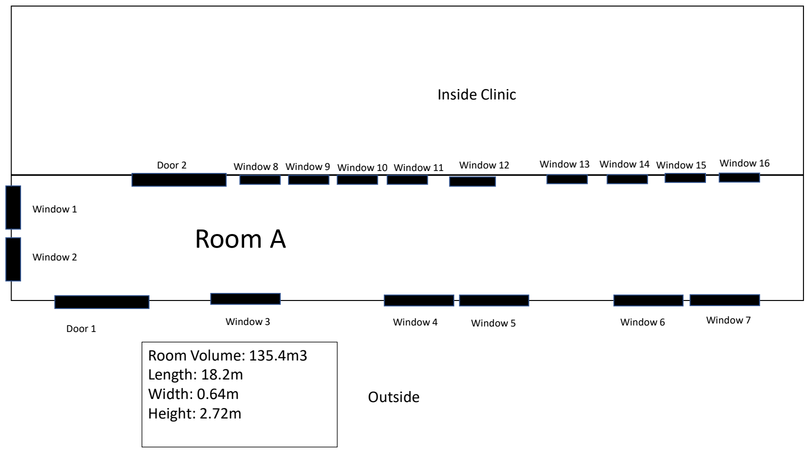

5

Room A: Dimensions

Window/Door	Facing	Type of window	Height	Width	Area (m2)	How it was open on day of observation
Window 1	Outside	Casement	1.18	0.88	1.04	Window closed
Window 2	Outside	Casement	1.18	0.88	1.04	Window closed
Window 3	Outside	Casement	1.18	0.88	1.04	Full open
Window 4	Outside	Casement	1.18	0.88	1.04	10 degrees
Window 5	Outside	Casement	1.18	0.88	1.04	10 degrees
Window 6	Outside	Casement	1.18	0.88	1.04	Fully Open
Window 7	Outside	Casement	1.18	0.88	1.04	Fully
Window 8	Into clinic	Casement	1.18	0.47	0.55	Closed
Window 9	Into clinic	Awning	0.29	0.47	0.14	Closed
Window 10	Into Clinic	Awning	0.29	0.47	0.14	45 degrees
Window 11	Into clinic	Casement	1.18	0.47	0.55	45 degrees
Window 12	Into clinic	Casement	0.9	0.47	0.42	10 degrees
Window 13	Into clinic	Awning	0.59	0.47	0.28	Closed
Window 14	Into clinic	Awning	0.59	0.47	0.28	Closed
Window 15	Into clinic	Casement	0.59	0.47	0.28	Closed
Window 16	Into Clinic	Casement	0.9	0.47	0.42	5 degrees
Door 1	Outside	Double door	2.0	1.59	3.22	Fully Open
Door 2	Into clinic	Double door	1.96	1.8	3.52	Fully Open

6

Room C

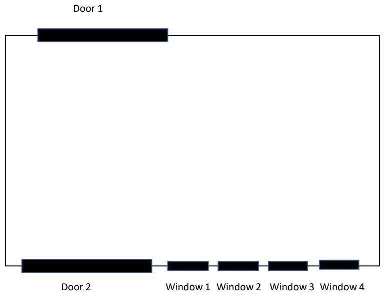

Room Volume: 42.68m3
Length: 4.331m
Width: 3.77m
Height: 2.62

7

Room C dimensions

Window/Door	Facing	Type of window/window	Height	Width	Area (m2)	How it was open on day of observation
Window 1	Into waiting area	Casement	1.18	0.47	0.55	Closed
Window 2	Into waiting area	Casement	0.29	0.47	0.14	Closed
Window 3	Into waiting area	Casement	0.29	0.47	0.14	45 degrees
Window 4	Into waiting area	Casement	1.18	0.47	0.55	45 degrees
Door 1	Into clinic	Double door	1.98	1.59	3.15	Fully Open
Door 2	Into clinic	Double door	1.96	1.8	3.52	Fully Open

8

Room H

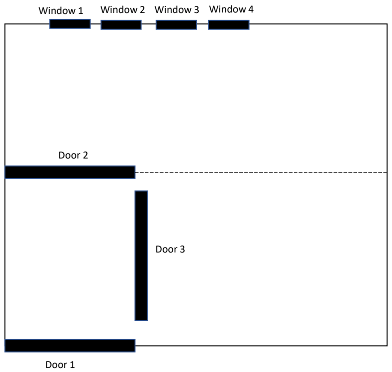

Room Volume: 42.68m3
Length: 4.161m
Width: 3.766m
Height: 2.6m

9

Room H dimensions

Window/Door	Type of window	Height	Width	Area (m2)	How it was open on day of observation
Window 1	Casement	1.19	0.47	0.55	20 degrees
Window 2	Awning	0.91	0.28	0.26	20 degrees
Window 3	Awning	0.91	0.28	0.26	Closed
Window 4	Casement	1.19	0.47	0.55	Closed
Door 1	Single door	2.03	0.81	1.66	Open
Door 2	Single door	2.03	0.81	1.66	Closed
Door 3	Single door	2.03	0.81	1.66	Closed

10

Room K

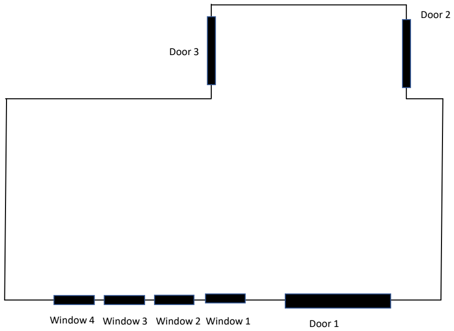

Room Volume:
Length: 4.161m
Width: 3.766m
Height: 2.6m

11

Room K dimensions

Window/Door	Type of window	Height	Width	Area (m2)	How it was open on day of observation
Window 1	Casement	0.88	0.44	0.39	5 degrees
Window 2	Awning	0.26	0.91	0.24	5 degrees
Window 3	Casement	0.88	0.44	0.39	Closed
Window 4	Awning	0.26	0.91	0.24	5 degrees
Door 1	Double door	2.00	1.60	3.20	1 door open of the two (half the door open)
Door 2	Single door	2.02	0.82	1.64	Closed
Door 3	Single door	2.00	0.82	1.64	Closed

12

Room M

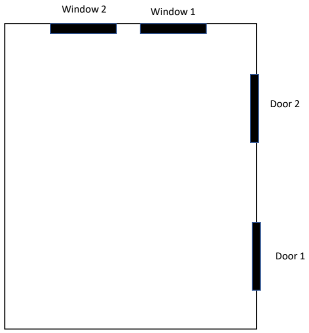

Room Volume: 44.78 m3
Length: 5.15m
Width: 3.23m
Height: 2.69m

13

Room M Dimensions

Window/Door	Type of window	Height	Width	Area (m2)	How it was open on day of observation
Window 1	Casement	0.88	0.46	0.40	Closed
Window 2	Awning	0.27	0.93	0.25	Fully open (45 degrees)
Door 1	Single	2.01	0.81	1.63m3	Door closed
Door 2	Single	2.01	0.81	1.63m3	Door closed

14

Room S

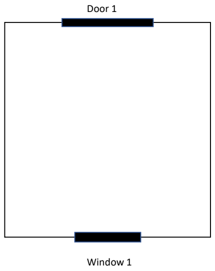

Room Volume: 14.69m3
Length: 2.86m
Width: 2.10m
Height: 2.45m

Door 1
Height: 2.04m
Width: 0.82m
Under usual circumstances:
Door closed

Window: Sliding
Height: 0.84m
Width: 0.55m
Under usual circumstances:
Slid open by 20cm

15

16

Room V

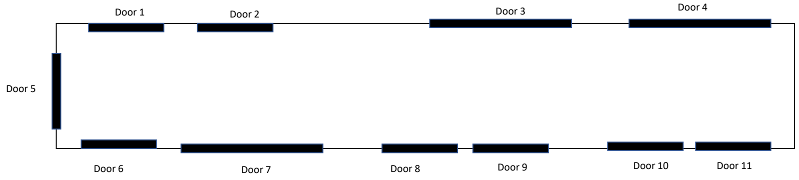

Room Volume: 20.42m3
Length: 14.7m
Width: 1.38m
Height: 2.61m

Dimensions of Room V

Door	Height	Width	Area (m2)	How it was open on day of observation
Door 1	2.03	0.81	1.66	Closed
Door 2	2.03	0.81	1.66	Open
Door 3	1.97	1.59	3.13	Closed
Door 4	1.97	1.59	3.13	One half open
Door 5	2.044	0.82	1.68	Closed
Door 6	2.03	0.81	1.66	Closed
Door 7	1.96	1.8	3.52	Fully Open
Door 8	2.03	0.81	1.66	Open
Door 9	2.03	0.81	1.66	Closed
Door 10	2.03	0.81	1.66	Closed
Door 11	2.03	0.81	1.66	Closed

HAST waiting area

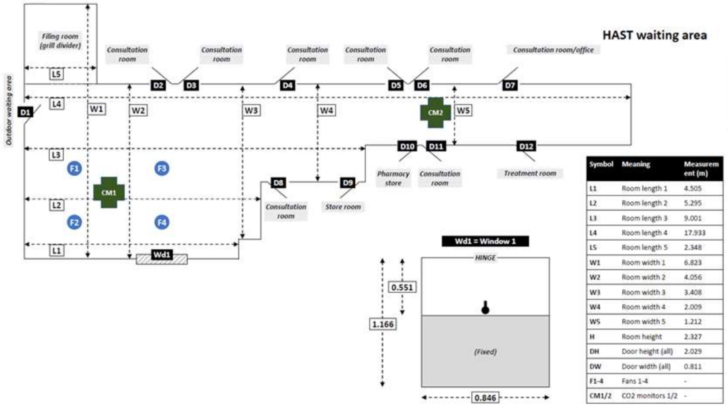

Symbol	Meaning	Measurement (m)
L1	Room length 1	4.505
L2	Room length 2	5.295
L3	Room length 3	9.001
L4	Room length 4	17.933
L5	Room length 5	2.348
W1	Room width 1	6.823
W2	Room width 2	4.056
W3	Room width 3	3.408
W4	Room width 4	2.009
W5	Room width 5	1.212
H	Room height	2.327
DH	Door height (all)	2.029
DW	Door width (all)	0.811
F1-4	Fans 1-4	-
CM1/2	CO2 monitors 1/2	-

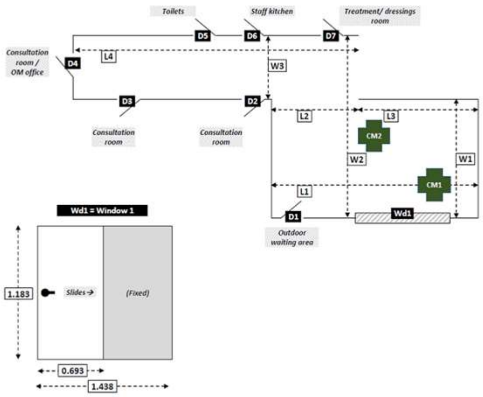

TB waiting area

Symbol	Meaning	Measurement (m)
L1	Room length 1	4.795
L2	Room length 2	1.275
L3	Room length 3	3.553
L4	Room length 4	5.753
W1	Room width 1	3.683
W2	Room width 2	4.588
W3	Room width 3	1.218
W4	Room width 4	2.402
H	Room height	2.402
DH	Door height (all)	2.036
DW	Door width (all)	0.918
CM1/2	CO2 monitors 1/2	-

Space L (main indoor waiting area)

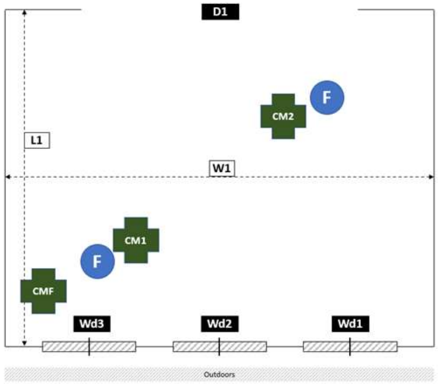

Symbol	Meaning	Measurement (m)
**L1**	Length of room	5.876
**W1**	Width of room	3.339
**H1**	Height of room	2.558
**D1**	**Doorway (no door)**	-
**D1W**	Width of doorway D1	2.028
**D1H**	Height of doorway D1	2.048
**Wd1/2/3**	**Windows 1/2/3**	-
**WdH**	Height of windows Wd1/2/3	0.856
**WdW**	Width of windows Wd1/2/3	0.909
**F**	Ceiling fan	-
**CMF**	CO2 meter during patient flow	-
**CM1**	CO2 meter 1 during CO2 release	-
**CM2**	CO2 meter 2 during CO2 release	-

21

Spaces S/T/U

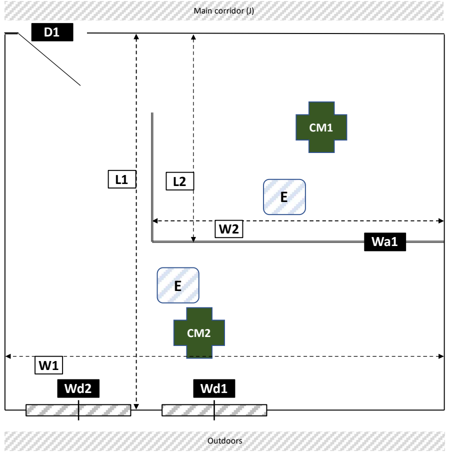

Symbol	Meaning	Measurement (m)
**L1**	Room length 1	5.868
**L2**	Room length 2 (bloods area)	2.961
**W1**	Room width 1	3.458
**W2**	Room width 2	1.958
**H**	Room height	2.553
**Wa1**	Divider wall (does not reach ceiling)	-
**Wa1H**	Height of divider wall	2.109
**D1**	**Doorway 1 (single door)**	-
**D1H**	Height of doorway 1	2.004
**D1W**	Width of doorway 1	0.978
**Wd1/2**	**Windows 1/2 (both double)**	-
**WdH**	Height of windows 1/2	0.863
**WdW**	Width of windows 1/2	0.849
**E**	Ceiling extractor/air conditioner	-
**CM1**	CO2 meter 1 during CO2 release	-
**CM2**	CO2 meter 2 during CO2 release	-

22

Space K/J

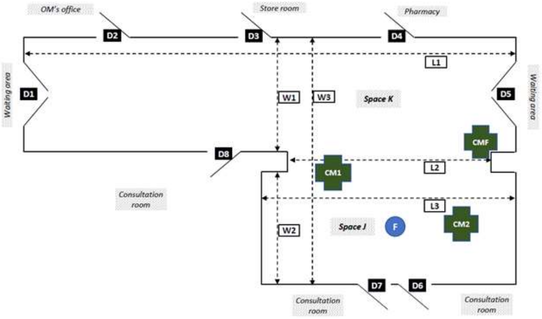

Symbol	Meaning	Measurement (m)
**L1**	Length of space K	7.975
**L2**	Length of 'doorway' K/J	5.179
**L3**	Length of space J	5.785
**W1**	Width of space K	1.973
**W2**	Width of space J	1.764
**W3**	Width of space K/J	4.009
**H**	Height (throughout spaces K & J)	2.498
**F**	Ceiling fan	-
**CM1/2**	CO2 monitor 1/2	-
**CMF**	CO2 monitor during patient flow	-
**D1**	**Double doors (also D5)**	-
**D1H**	Double door height	2.054
**D1W**	Double door width	1.594
**D2**	**Single door (also D3,D4, & D6-D8)**	-
**D2H**	Single door height	2.028
**D2W**	Single door width	0.882

23

Space N

(designed for TB, used for vaccinations)

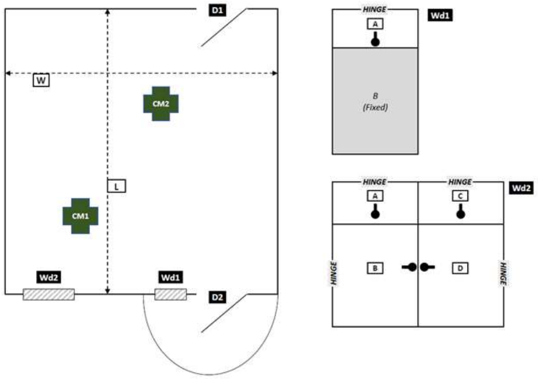

Symbol	Meaning	Measurement (m)
**L**	Length of space N	3.810
**W**	Width of space N	3.772
**H**	Height of space N	2.506
**CM1/2**	CO2 monitor 1/2	-
**D1**	**Single internal door**	-
**D1H**	D1 height	2.043
**D1W**	D1 width	0.870
**D2**	**Single external door**	-
**D2H**	D2 height	2.002
**D2W**	D2 width	0.783
**Wd1**	**Window 1**	-
**Wd1TH**	Wd1 total height	1.006
**Wd1TW**	Wd1 total width	0.446
**Wd1AH**	Wd1 section A height	0.402
**Wd2**	**Window 2**	-
**Wd2TH**	Wd2 total height	1.473
**Wd2TW**	Wd2 total width	0.982
**Wd2ABW**	Wd2 section A/B width	0.486
**Wd2AH**	Wd2 section A height	0.586
**Wd2BH**	Wd2 section B height	0.877

24

Room CR3

Room Volume: 30.95m3
Length: 3.69m
Width: 3.02m
Height: 2.78m

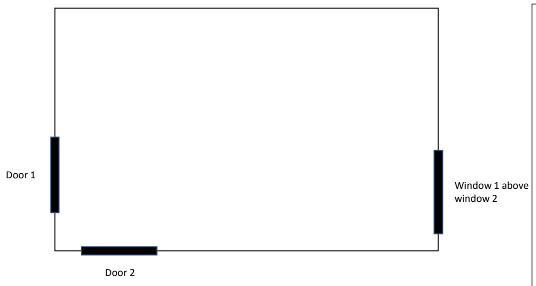

Door 1
Height: 2.36 m
Width: 0.79m
Door 2
Height: 2.36m
Width: 0.79m
Under usual circumstances:
Both doors closed
Window 1: Awning
Height: 0.34m
Width: 1.10m
Closed under usual
Window 2: Awning
Height: 0.65m
Width: 1.01m
Under usual circumstances
Open: 10 degrees

Room CR1

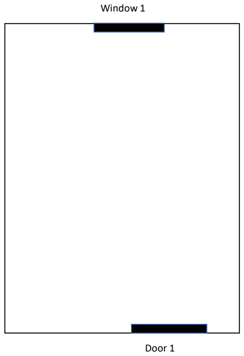

Room Volume: 29.37m3
Length: 4.00m
Width: 2.99m
Height: 2.48m

Door 1
Height: 2.01 m
Width: 0.78 m
Under usual circumstances:
Door closed
Window 1: Casement
Height: 0.96m
Width: 0.88
Under usual circumstances:
Open by 30 degrees

Room D

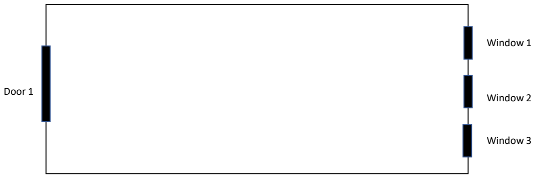

Room Volume: 22.38 m3
Length: 3.99m
Width: 2.19m
Height: 2.59m

Room D Dimensions

Door	Type of window/door	Height	Width	Area (m2)	How it was open on day of observation
Window 1	Casement	0.88	0.47	0.41	Closed
Window 2	Awning	0.29	0.47	0.14	Closed
Window 3	Casement	0.88	0.47	0.41	Closed
Door 1	Single	2.00	0.79	0.82	Closed

Room G

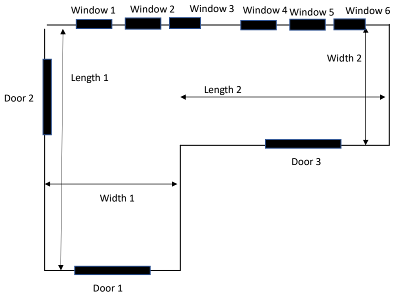

Room Volume: 31.26 m3

Length 1: 3.99 m
Width 1: 2.36 m

Length 2: 2.11m
Width 2: 1.51m
Height: 2.54 m

29

Room G dimensions

Door	Type of window/door	Height	Width	Area (m2)	How it was open on day of observation
Window 1	Casement	0.88	0.47	0.41	Maximally open (Protective change limiting it to 30 degrees
Window 2	Awning	0.29	0.47	0.14	Maximally open (45 degress limited by protective cage)
Window 3	Casement	0.88	0.47	0.41	Maximally open (30 degrees secondary to change
Window 4	Casement	0.88	0.47	0.41	Maximally open (Protective change limiting it to 30 degrees
Window 5	Awning	0.29	0.47	0.14	Maximally open (45 degress limited by protective cage)
Window 6	Casement	0.88	0.47	0.41	Maximally open (30 degrees secondary to change
Door 1	Single	2.00	0.79	0.82	Open
Door 2	Single	2.00	0.79	0.82	Closed
Door 3	Single	2.00	0.79	0.82	Closed

30

Room H

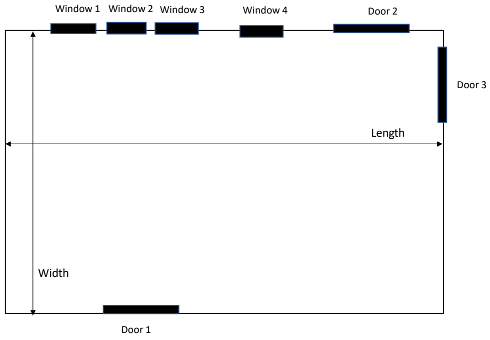

Room Volume: 45.46 m3

Length : 4.458 m
Width : 4.00m
Height: 2.55m

31

Room H dimensions

Door	Type of window/door	Height	Width	Area (m2)	How it was open on day of observation
Window 1	Casement	0.88	0.47	0.41	Maximally open 30 degrees
Window 2	Awning	0.29	0.47	0.14	Maximally open (45 degrees limited by protective cage)
Window 3	Casement	0.88	0.47	0.41	Maximally open: 30 degrees
Window 4	Casement	0.88	0.47	0.41	Maximally open to 30 degrees
Door 1	Single	2.00	0.79	0.82	Closed
Door 2	Single	2.00	0.79	0.82	Closed
Door 3	Single	2.00	0.79	0.82	Closed

32

Room J

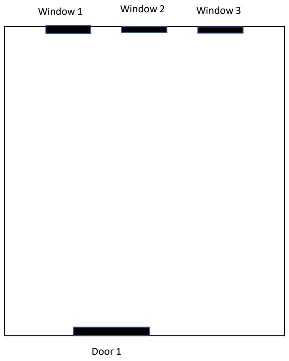

Room Volume: 22.09m3

Length : 4.00 m
Width : 2.165m
Height: 2.55m

Room J dimensions

Door	Type of window/door	Height	Width	Area (m2)	How it was open on day of observation
Window 1	Casement	0.88	0.47	0.41	Maximally open 30 degrees
Window 2	Awning	0.29	0.47	0.14	Closed
Window 3	Casement	0.88	0.47	0.41	Maximally open: 30 degrees
Door 1	Single	2.00	0.79	0.82	Closed

Room A

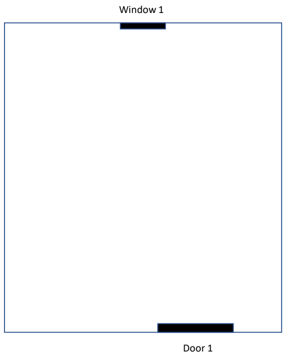

Room Volume: 32.86m3

Length : 4.52 m
Width : 2.65 m
Height: 2.731m

Door 1
Length: 2.02m
Width: 0.78m
Area: 1.58m2
Under usual conditions: door closed

Window 1: Hopper type window
Height: 0.66m
Width: 0.64m
Area: 0.42m2
Under usual conditions: window maximally open: 30 degrees open
